# ADAMTS-4 promotes neurodegeneration in a mouse model of amyotrophic lateral sclerosis

**DOI:** 10.1186/s13024-016-0078-3

**Published:** 2016-01-25

**Authors:** Sighild Lemarchant, Yuriy Pomeshchik, Iurii Kidin, Virve Kärkkäinen, Piia Valonen, Sarka Lehtonen, Gundars Goldsteins, Tarja Malm, Katja Kanninen, Jari Koistinaho

**Affiliations:** Department of Neurobiology, A. I. Virtanen Institute for Molecular Sciences, Biocenter Kuopio, University of Eastern Finland, P.O. Box 1627, 70211 Kuopio, Finland

**Keywords:** A desintegrin and metalloproteinase with thrombospondin motifs, Amyotrophic lateral sclerosis, Extracellular matrix, Neurodegeneration, Perineuronal net, Chondroitin sulfate proteoglycan, Astrogliosis, Nerve growth factor, Brain-derived neurotrophic factor, Glial cell-derived neurotrophic factor

## Abstract

**Background:**

A disintegrin and metalloproteinase with thrombospondin motifs (ADAMTS) proteoglycanases are specialized in the degradation of chondroitin sulfate proteoglycans and participate in mechanisms mediating neuroplasticity. Despite the beneficial effect of ADAMTS-4 on neurorepair after spinal cord injury, the functions of ADAMTS proteoglycanases in other CNS disease states have not been studied. Therefore, we investigated the expression, effects and associated mechanisms of ADAMTS-4 during amyotrophic lateral sclerosis (ALS) in the SOD1^G93A^ mouse model.

**Results:**

ADAMTS-4 expression and activity were reduced in the spinal cord of SOD1^G93A^ mice at disease end-stage when compared to WT littermates. To counteract the loss of ADAMTS-4, SOD1^G93A^ and WT mice were treated with saline or a recombinant ADAMTS-4 before symptom onset. Administration of ADAMTS-4 worsened the prognosis of SOD1^G93A^ mice by accelerating clinical signs of neuromuscular dysfunctions. The worsened prognosis of ADAMTS-4-treated SOD1^G93A^ mice was accompanied by increased degradation of perineuronal nets enwrapping motoneurons and increased motoneuron degeneration in the lumbar spinal cord. Motoneurons of ADAMTS-4-treated SOD1^G93A^ mice were more vulnerable to degeneration most likely due to the loss of their extracellular matrix envelopes. The decrease of neurotrophic factor production induced by ADAMTS-4 in vitro and in vivo may also contribute to a hostile environment for motoneuron especially when devoid of a net.

**Conclusions:**

This study suggests that the reduction of ADAMTS-4 activity during the progression of ALS pathology may be an adaptive change to mitigate its neurodegenerative impact in CNS tissues. Therapies compensating the compromized ADAMTS-4 activity are likely not promising approaches for treating ALS.

## Background

A disintegrin and metalloproteinase with thrombospondin motifs type 4, ADAMTS-4, belongs to the subfamily of ADAMTS proteases capable of degrading proteoglycans. The subfamily is composed of ADAMTS-1, −4, −5, −8, −9, −15 and −20 [1, 2]. Increasing evidence suggests that some ADAMTS proteoglycanases, for instance ADAMTS-1 and −4, may play critical roles in the control of synaptic plasticity during CNS development and aging via both protease-dependent and independent mechanisms [2–4]. In addition, administration of ADAMTS-4 has been recently described as a promising therapeutic strategy to improve axonal regeneration/collateral sprouting after spinal cord injury in rats by degrading chondroitin sulfate proteoglycans [5, 6]. While deregulated expression of ADAMTS proteoglycanases has been previously reported during acute CNS injuries, such as stroke [7–9] and spinal cord injury [5, 6, 10], the expression and function of ADAMTS proteoglycanases have not been studied in neurodegenerative diseases, such as amyotrophic lateral sclerosis (ALS).

ALS is a devastating neurodegenerative disease characterized by the selective death of upper and lower motoneurons. Muscle wasting and weakness are early signs of ALS, and finally, the patient’s death occurs usually within 3–5 years after disease onset. In 90 % of ALS cases, no apparent familial linkage has been identified, but in the remaining 10 % of the patients, the disease is inherited [11]. Autosomal dominant mutations in the *Cu, Zn-superoxide dismutase* (*SOD1*) gene account for 20 % of the familial disease form [12, 13]. The two forms of ALS are clinically indistinguishable and share many pathogenic features including oxidative damage, mitochondrial dysfunction, endoplasmic reticulum stress, excitotoxicity and inflammation [14]. Riluzole is the only FDA-approved drug for the treatment of ALS but it unfortunately has a modest impact of prolonging the life span of patients by only 2–3 months [15]. Therefore, it is essential to further understand mechanisms underlying ALS development in order to find new approaches for diagnostics and therapy.

Considering the beneficial effect of ADAMTS-4 on neuroplasticity, we aimed at investigating the expression, effects and associated mechanisms of ADAMTS-4 in ALS. While the expressions of ADAMTS-1, −5 and −9 were increased in the lumbar spinal cord of SOD1^G93A^ mice compared to corresponding WT littermates, the expression and activity of the most expressed proteoglycanase, ADAMTS-4, were reduced at the end-stage of the disease. To counteract the loss of ADAMTS-4 expression in the spinal cord, recombinant ADAMTS-4 was administered to SOD1^G93A^ mice early prior to the onset of symptoms by intracerebroventricular injections. Surprisingly, ADAMTS-4 treatment promoted the degeneration of lumbar spinal motoneurons by degrading their perineuronal nets and led to a detrimental functional outcome in SOD1^G93A^ mice. Our results also show that ADAMTS-4 decreased the synthesis and release of neurotrophic factors by astrocytes and microglia in vitro and in vivo.

While ADAMTS-4 has a beneficial impact on neuroplasticity and the subsequent functional outcome of injured rats after spinal cord injury, it may represent a damageable target in the context of ALS by accelerating neurodegeneration and clinical signs of neuromuscular dysfunctions in the SOD1^G93A^ mouse model. The modulation of the synthesis and release of neurotrophic factors by endogenous or exogenous ADAMTS-4 shows that ADAMTS-4 functions are not limited solely to the degradation of the extracellular matrix.

## Results

### ADAMTS-4 is the most expressed ADAMTS proteoglycanase in the central nervous system

**Fig. 1:**
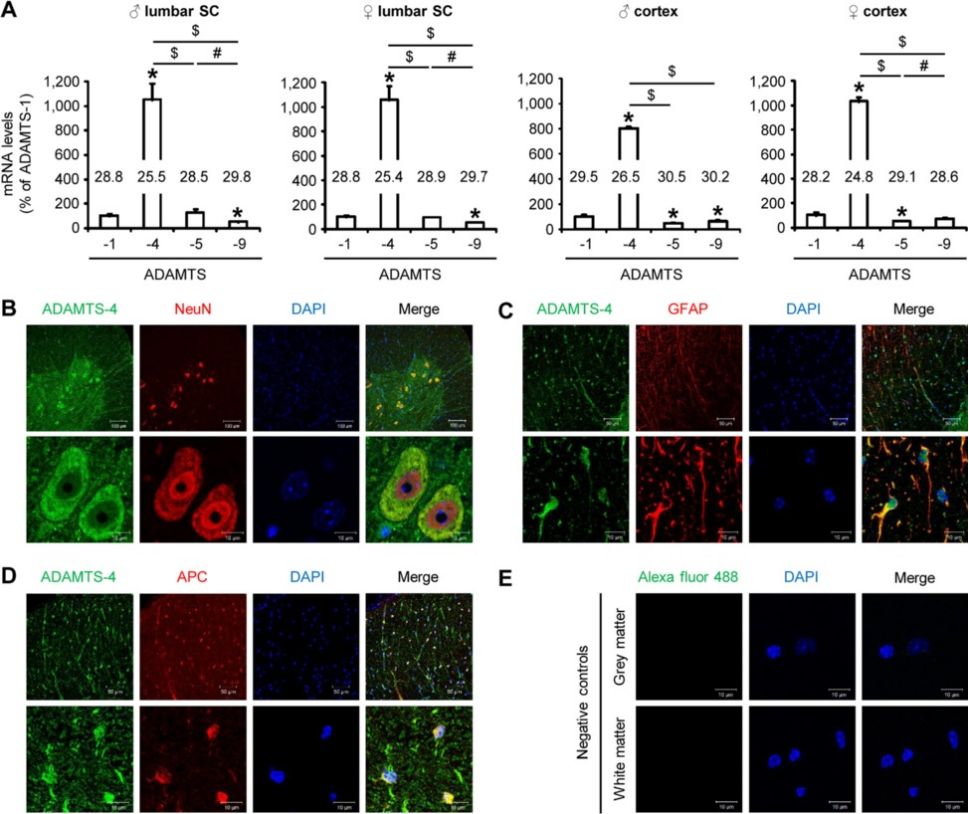
ADAMTS-4 expression in the central nervous system. **a** Differential mRNA expression of ADAMTS proteoglycanases (eg. ADAMTS-1, −4, −5 and −9) in the lumbar spinal cord (SC) and in the cortex of 3-month-old WT male (♂) and female (♀) mice. Ct values are indicated in the histograms. Values plotted are mean ± SEM. Mann–Whitney *U*-tests: ^*^ *P <* 0.05 compared to ADAMTS-1 expression, ^$^ *P <* 0.05 compared to ADAMTS-4 expression, ^#^ *P <* 0.05 compared to ADAMTS-5 expression, *N* = 3-4. **b-d** Representative photomicrographs of lumbar spinal cord sections from WT mice stained with: ADAMTS-4 (green) and NeuN (neuronal marker, red) (**b**) ADAMTS-4 (green) and GFAP (astrocyte marker, red) (**c**) or ADAMTS-4 (green) and APC (oligodendrocyte marker, red) (**d**). Corresponding Alexa fluor-488 negative controls for ADAMTS-4 (green) in the grey and white matter are shown in **e**

We first studied the differential expression of ADAMTS proteoglycanases (ADAMTS-1, −4, −5, −9) in the lumbar spinal cord and in the cortex of adult WT mice by RT-PCR. ADAMTS-4 was at least 8-fold more expressed than the other ADAMTS proteoglycanases in the spinal cord and in the cortex of WT mice (Fig. 1a: *P* = 0.0209 and *P* = 0.0495, respectively). Confocal imaging revealed that the expression of ADAMTS-4 was widespread within the spinal cord in the grey matter and also in the white matter. Its expression was particularly abundant in ventral horn neurons (Fig. 1b), astrocytes (Fig. 1c) and oligodendrocytes (Fig. 1d). Negative controls with only the secondary antibody used for ADAMTS-4 staining failed to reveal any fluorescence (Fig. 1e).

### Decrease of ADAMTS-4 activity in the lumbar spinal cord of SOD1^G93A^ mice at disease end-stage

**Fig. 2:**
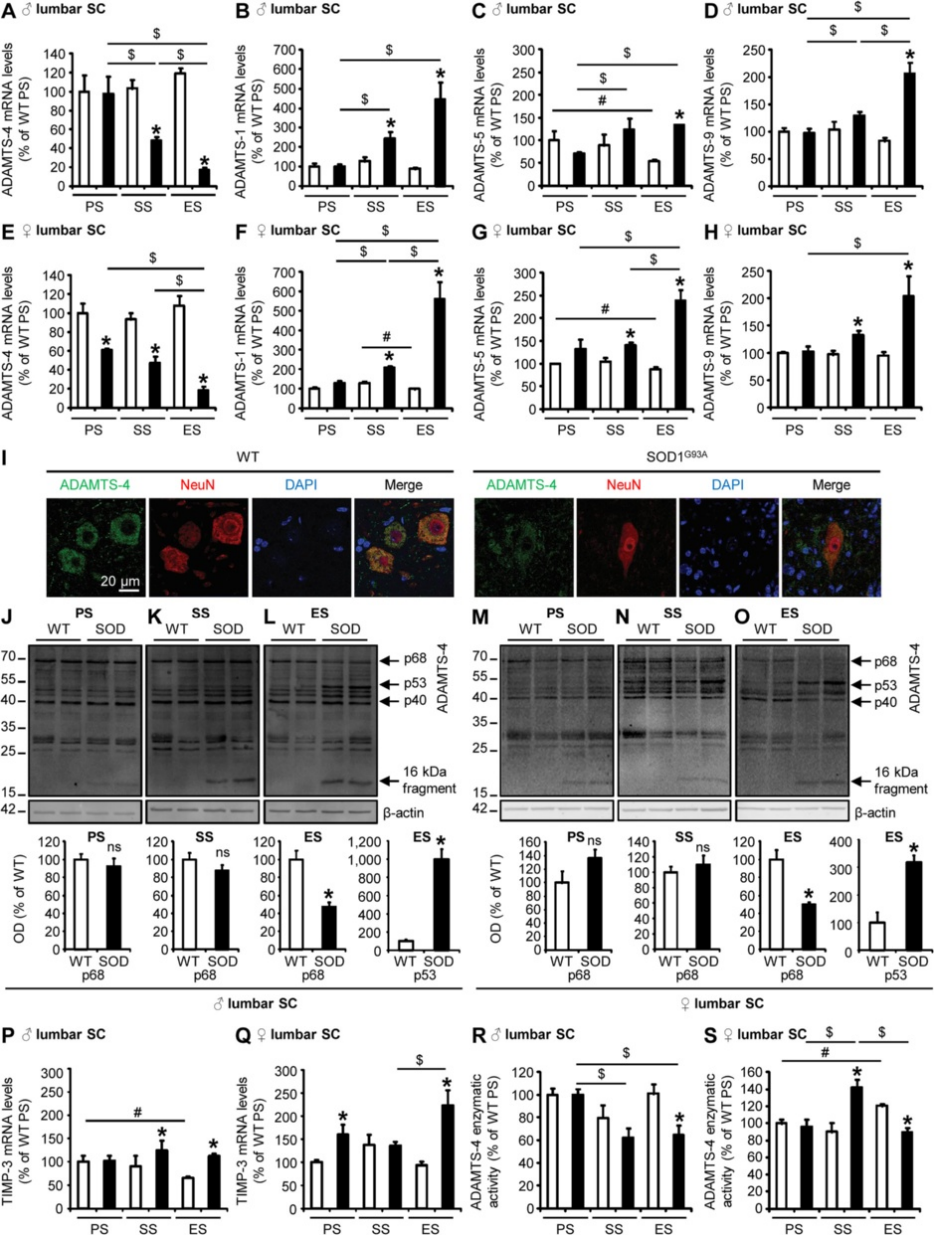
Decrease of ADAMTS-4 activity in the lumbar spinal cord of SOD1^G93A^ mice at disease end-stage. **a**-**h** Quantitative RT-PCR for ADAMTS-4 (**a**, **e**) ADAMTS-1 (**b**, **f**) −5 (**c**, **g**) and −9 (**d**, **h**) expression in the lumbar spinal cord (SC) of WT (blank bar) and SOD1^G93A^ (black bar) male (♂; **a**, **b**, **c**, **d**) or female (♀; **e**, **f**, **g**, **h**) mice at presymptomatic (PS), symptomatic (SS) and end (ES) stages. Values plotted are mean ± SEM. Mann–Whitney *U*-tests: ^*^ *P <* 0.05 compared to corresponding WT mice, ^$^ *P <* 0.05 compared to SOD1^G93A^ mice at other stages, ^#^ *P <* 0.05 compared to WT mice at other stages, *N* = 3-4. **i** Representative photomicrographs of ventral horns in lumbar spinal cord sections from WT and SOD1^G93A^ mice at end-stage stained with ADAMTS-4 (green) and NeuN (neuronal marker, red). Scale bar = 20 μm. **j**-**o** Immunoblot for ADAMTS-4 in the lumbar spinal cord of WT and SOD1^G93A^ male (**j**-**l**) and female (**m**-**o**) mice at PS, SS and ES. The immunoblots revealed ADAMTS-4 mature form (p68), ADAMTS-4 truncated forms (p53, p40) and a 16 kDa fragment. Values plotted are mean ± SEM. Mann–Whitney *U*-tests: ^*^ *P <* 0.05 compared to corresponding WT mice, *N* = 4-5. **p**-**q** Quantitative RT-PCR for TIMP-3 in the lumbar spinal cord of WT and SOD1^G93A^ male (**p**) and female (**q**) mice at PS, SS and ES. Values plotted are mean ± SEM. Mann–Whitney *U*-tests: ^*^ *P <* 0.05 compared to corresponding WT mice, ^$^ *P <* 0.05 compared to SOD1^G93A^ mice at other stages, ^#^ *P <* 0.05 compared to WT mice at other stages, *N* = 3-4. **r**-**s** ADAMTS-4 enzymatic activity assay in the lumbar spinal cord of WT and SOD1^G93A^ male (**r**) and female (**s**) mice at PS, SS and ES. Values plotted are mean ± SEM. Mann–Whitney *U*-tests: ^*^ *P <* 0.05 compared to corresponding WT mice, ^$^ *P <* 0.05 compared to SOD1^G93A^ mice at other stages, ^#^ *P <* 0.05 compared to WT mice at other stages, *N* = 4

We next studied the time course of the expression of ADAMTS proteoglycanases (ADAMTS-1, −4, −5, −9) in the lumbar spinal cord of SOD1^G93A^ and age-matched WT mice by RT-PCR at key time points of ALS progression (*eg*. presymptomatic (PS), symptomatic (SS) and end (ES) stages). ADAMTS-4 mRNA levels were considerably decreased in SOD1^G93A^ male mice compared to WT at the symptomatic and end-stages of the disease (Fig. 2a: −53.7 % at SS, −85.7 % at ES compared to age-matched WT, *P* = 0.0209). Contrary to ADAMTS-4, ADAMTS-1 (Fig. 2b: +92.1 % at SS, +410.7 % at ES compared to age-matched WT, *P* = 0.0433, *P* = 0.0209, respectively), ADAMTS-5 (Fig. 2c: +148.9 % at ES compared to age-matched WT, *P* = 0.0339) and ADAMTS-9 (Fig. 2d: +149.6 % at ES compared to age-matched WT, *P* = 0.0209) mRNA levels were significantly increased in the lumbar spinal cord of SOD1^G93A^ male mice compared to WT at the symptomatic and/or end-stages of the disease. Similarly, ADAMTS-4 mRNA levels were considerably decreased in the lumbar spinal cord of SOD1^G93A^ female mice compared to WT at all the stages of the disease (Fig. 2e: −38.9 % at PS, −48.9 % at SS, −82.7 % at ES compared to age-matched WT, *P* = 0.0339, *P* = 0.0209, *P* = 0.0339, respectively). Conversely, ADAMTS-1 (Fig. 2f: +60.5 % at SS, +472 % at ES compared to age-matched WT, *P* = 0.0209, *P* = 0.0339, respectively), ADAMTS-5 (Fig. 2g: +33.6 % at SS, +171 % at ES compared to age-matched WT, *P* = 0.0209, *P* = 0.0339, respectively) and ADAMTS-9 (Fig. 2h: +35.8 % at SS, +114,9 % at ES compared to age-matched WT, *P* = 0.0209, *P* = 0.0339, respectively) mRNA levels were significantly increased in the lumbar spinal cord of SOD1^G93A^ female mice compared to WT at the symptomatic and end-stages of the disease. Confocal imaging revealed an abundant expression of ADAMTS-4 in ventral horn neurons of WT mice (Fig. 1b), and a loss/degeneration of motoneurons occurring at disease end-stage in SOD1^G93A^ mice (Fig. 2i).

No modifications of ADAMTS-4 protein levels were observed between WT and SOD1^G93A^ male mice at the presymptomatic and symptomatic stages (Fig. 2j-k: *P* = 0.1482 and *P* = 0.5637, respectively). However, at the end-stage of the disease, the decrease of ADAMTS-4 mRNA levels in the lumbar spinal cord of SOD1^G93A^ male mice was accompanied by a decrease of the protein levels of the mature form of ADAMTS-4 (p68) (Fig. 2l: −50.1 % at ES compared to age-matched WT, *P* = 0.0209) and an increase of its truncated form (p53) (Fig. 2l: +898.5 % at ES compared to age-matched WT, *P* = 0.0209). As previously observed for SOD1^G93A^ male mice, no modifications of ADAMTS-4 protein levels were observed between WT and SOD1^G93A^ female mice at the presymptomatic and symptomatic stages (Fig. 2m-n: *P* = 0.5637 and *P* = 0.2482, respectively), but at the end-stage of the disease, the decrease of ADAMTS-4 mRNA levels in the lumbar spinal cord of SOD1^G93A^ female mice was accompanied by a decrease of the protein levels of the mature form of ADAMTS-4 (p68) (Fig. 2o: −52.4 % at ES compared to age-matched WT, *P* = 0.0143) and an increase of its truncated form (p53) (Fig. 2o: +218 % at ES compared to age-matched WT, *P* = 0.0209). No significant modifications of ADAMTS-4 truncated form (p40) were observed (Figs. 2l, o: quantifications not shown).

Then, quantitative RT-PCR was performed for the mRNA expression of the most potent inhibitor of ADAMTS-4, TIMP-3 (type 3 tissue inhibitor of metalloproteinases) [16], in the lumbar spinal cord of WT and SOD1^G93A^ male and female mice at the different stages of the disease. TIMP-3 mRNA levels were found to be increased in the lumbar spinal cord of SOD1^G93A^ male mice compared to WT at the symptomatic and end-stages of the disease (Fig. 2p: +38.3 % at SS, +70.8 % at ES compared to age-matched WT, *P* = 0.0339, *P* = 0.0209, respectively). For female mice, TIMP-3 mRNA levels were also increased in the lumbar spinal cord of SOD1^G93A^ mice compared to WT at the presymptomatic and end-stages of the disease (Fig. 2q: +59.6 % at PS, +138.8 % at ES compared to age-matched WT, *P* = 0.0339).

Consequently, the enzymatic activity of ADAMTS-4 was reduced in the lumbar spinal cord of SOD1^G93A^ male (Fig. 2r: −35.8 % at ES compared to age-matched WT, *P* = 0.0209) and female (Fig. 2s: −25.9 % at ES compared to age-matched WT, *P* = 0.0209) mice at disease end-stage

**Fig. 3:**
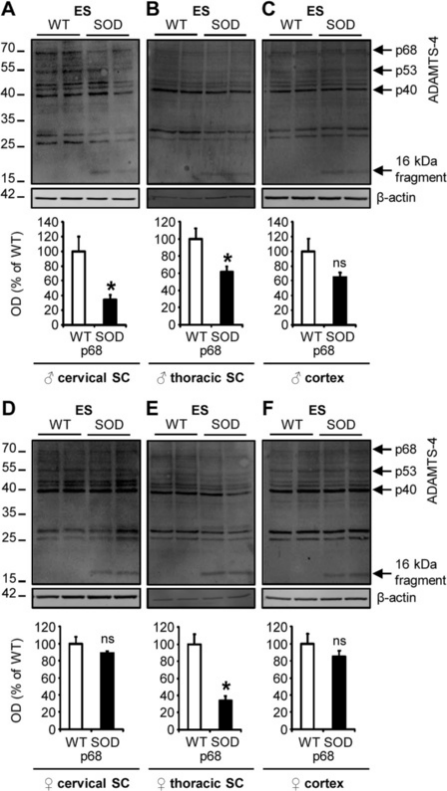
Spinal cord-specific decrease of ADAMTS-4 expression in SOD1^G93A^ mice at disease end-stage. **a-f** Immunoblot for ADAMTS-4 in the cervical or thoracic spinal cord (SC) and in the cortex of WT (blank bar) and SOD1^G93A^ (black bar) male (♂; **a**, **b**, **c**) or female (♀; **d**, **e**, **f**) mice at disease end-stage (ES). Values plotted are mean ± SEM. Mann–Whitney *U*-tests: ^*^ *P <* 0.05 compared to corresponding WT mice, *N* = 4

To further examine whether the loss of ADAMTS-4 at disease end-stage was specific to the lumbar spinal cord, we studied the expression of ADAMTS-4 in the cervical and thoracic parts of the spinal cord, as well as in the cortex, of WT and SOD1^G93A^ mice by western blot. Interestingly, there was a decrease of ADAMTS-4 expression in the cervical and thoracic spinal cord of SOD1^G93A^ male mice compared to WT (Fig. 3a-b: −65.5 % in the cervical (A) and −38.2 % in the thoracic (B) spinal cord at ES compared to age-matched WT, *P* = 0.0209), but no significant modification of ADAMTS-4 expression in the cortex was observed (Fig. 3c: *P* = 0.1489). For females, no modification of ADAMTS-4 expression was observed in the cervical spinal cord of SOD1^G93A^ mice compared to WT (Fig. 3d: *P* = 0.1489), but we observed a significant decrease of ADAMTS-4 expression in the thoracic spinal cord of SOD1^G93A^ mice compared to WT (Fig. 3e: −66.3 % in the thoracic spinal cord at ES compared to age-matched WT, *P* = 0.0209). Again, no modification of ADAMTS-4 expression was observed in the cortex (Fig. 3f: *P* = 0.2207). No significant modifications of ADAMTS-4 truncated forms (p53 and p40) were observed (Figs. 3a-f; quantifications not shown). We also observed the appearance of a 16 kDa fragment in protein extracts of both spinal cords and cortices of SOD1^G93A^ mice at all the stages of the disease, even when the mature form of ADAMTS-4 (p68) was unchanged when compared to corresponding WT mice (Fig. 2j-o, Fig. 3a-f).

**Fig. 4:**
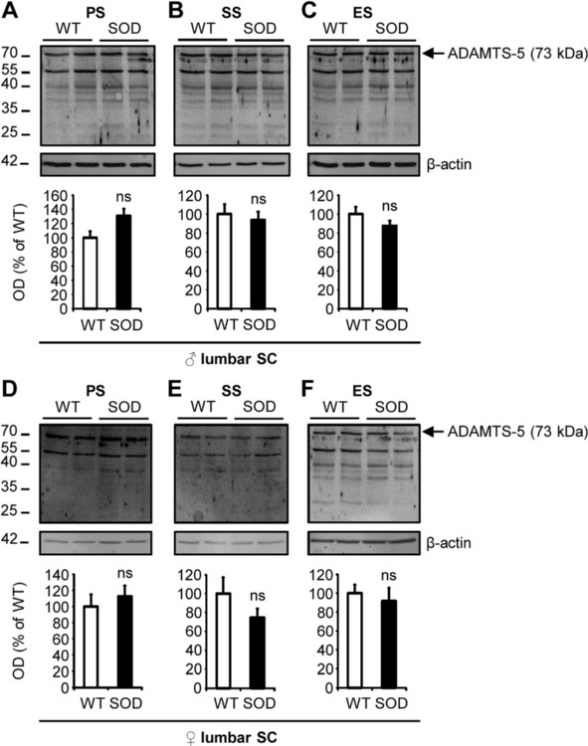
No modification of ADAMTS-5 expression in the lumbar spinal cord of SOD1^G93A^ mice at any stage of the disease. **a-f** Immunoblot for ADAMTS-5 (73 kDa) in the lumbar spinal cord of WT and SOD1^G93A^ male (**a-c**) and female (**d-f**) mice at PS, SS and ES. Values plotted are mean ± SEM. Mann–Whitney *U*-tests: *P >* 0.05 compared to corresponding WT mice, *N* = 4

Then, we studied the expression of ADAMTS-5 in the lumbar spinal cord of WT and SOD1^G93A^ mice by western blot. Surprisingly, while the mRNA levels of ADAMTS-5 were increased in the lumbar spinal cord at the end-stage in SOD1^G93A^ male mice and at the symptomatic and end-stages in SOD1^G93A^ female mice, no modifications were found at the protein levels between WT and SOD1^G93A^ mice at any stage of the disease (Fig. 4a-c: *P* = 0.0833, *P* = 0.3865, *P* = 0.2482 at PS, SS and ES respectively compared to age-matched male WT; Fig. 4d-f: *P* = 0.5637, *P* = 0.2482, *P* = 0.5637 at PS, SS and ES respectively compared to age-matched female WT).

To summarize, the expression and the synthesis of ADAMTS-4 and its inhibitor TIMP-3, as well as ADAMTS-4 proteolytic cleavage profile, were considerably altered in the spinal cord of SOD1^G93A^ mice at the end-stage of the disease, representing a series of events leading to the decrease of ADAMTS-4 enzymatic activity.

### Presymptomatic treatment with recombinant ADAMTS-4 worsens the prognosis of SOD1^G93A^ mice

**Fig. 5:**
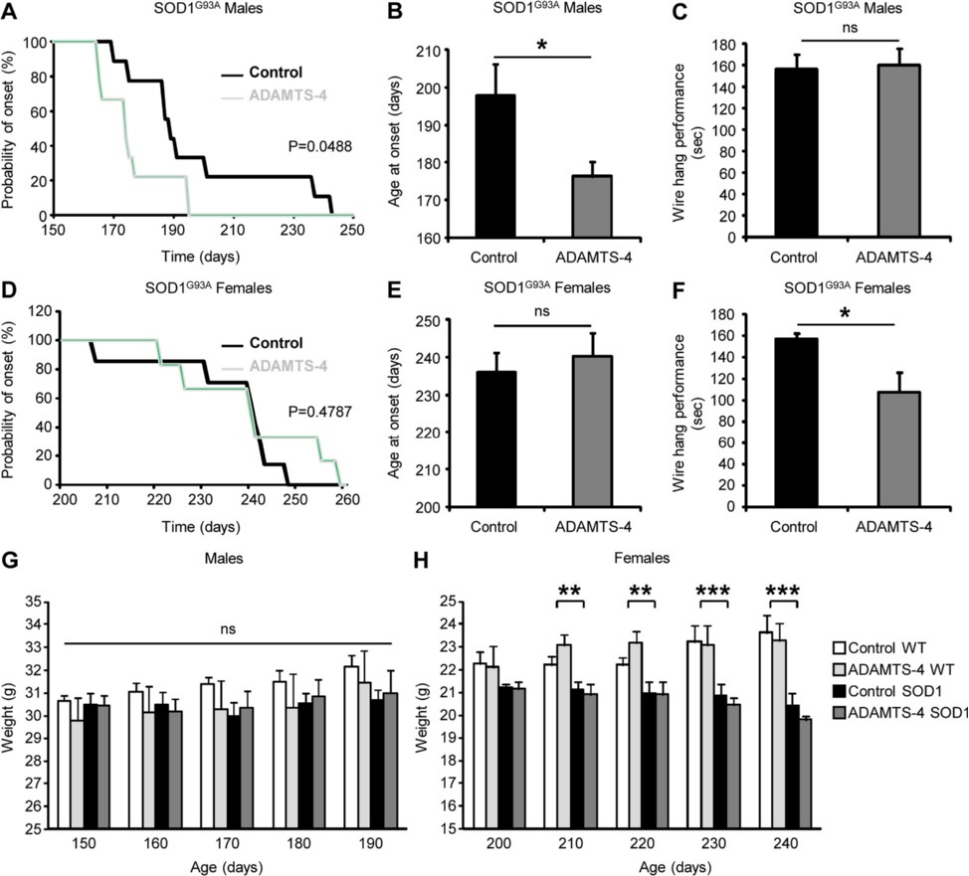
Presymptomatic treatment with rADAMTS-4 worsens the prognosis of SOD1^G93A^ mice. **a** Kaplan-Meier graph showing the probability of symptom onset in SOD1^G93A^ males treated with saline (Control; black line) or recombinant ADAMTS-4 (ADAMTS-4; gray line) at early presymptomatic stage. Log-rank (Mantel-Cox) Test: ^*^ *P <* 0.05 compared to the control group, *N* = 9. **b** Mean age at onset of mice from panel **a**. Values plotted are mean ± SEM. Mann–Whitney *U*-test: ^*^ *P <* 0.05 compared to the control group, *N* = 9. **c** The best wire hang performance during the first week after symptom onset. Values plotted are mean ± SEM. Mann–Whitney *U*-test: *P >* 0.05 compared to the control group, *N* = 9. **d** Kaplan-Meier graph showing the probability of symptom onset in SOD1^G93A^ females treated with saline (Control; black line) or recombinant ADAMTS-4 (ADAMTS-4; gray line) during early presymptomatic stage. Log-rank (Mantel-Cox) Test: *P >* 0.05 compared to the control group, *N* = 7 Control SOD1^G93A^, *N* = 6 ADAMTS-4 SOD1^G93A^. **e** Mean age at onset of mice from panel *D*. Values plotted are mean ± SEM. Mann–Whitney *U*-test: *P >* 0.05 compared to the control group, *N* = 7 Control SOD1^G93A^, *N* = 6 ADAMTS-4 SOD1^G93A^. **f** The best wire hang performance during the first week after symptom onset. Values plotted are mean ± SEM. Mann–Whitney *U*-test: ^*^ *P <* 0.05 compared to the control group, *N* = 7 Control SOD1^G93A^, *N* = 6 ADAMTS-4 SOD1^G93A^. **g**-**h** Weight over time of WT and SOD1^G93A^ male (**g**) or female (**h**) mice treated or not with saline or recombinant ADAMTS-4 during early presymptomatic stage. Values plotted are mean ± SEM. Two-way Anova: ^**^ *P <* 0.01, ^***^ *P <* 0.001 WT *Vs* SOD1^G93A^, *N* = 5 Control WT males, *N* = 5 ADAMTS-4 WT males, *N* = 9 Control SOD1^G93A^ males, *N* = 9 ADAMTS-4 SOD1^G93A^ males, *N* = 5 Control WT females, *N* = 4 ADAMTS-4 WT females, *N* = 7 Control SOD1^G93A^ females, *N* = 6 ADAMTS-4 SOD1^G93A^ females

To prevent the loss of ADAMTS-4 activity at disease end-stage, a human recombinant ADAMTS-4 previously shown to be biologically active and to support neuroplasticity [5] was administered to SOD1^G93A^ mice early before the onset of symptoms by intracerebroventricular injections. Control SOD1^G93A^ mice were injected with saline in the same conditions. Age-matched WT mice were also treated with saline or ADAMTS-4. The onset of symptoms was determined by the appearance of clinical signs of neuromuscular dysfunction, measured by the loss of ability for SOD1^G93A^ mice to hold onto an inverted lid. ADAMTS-4 treatment was detrimental in SOD1^G93A^ male mice by bringing forward the probability of onset of symptoms compared to saline-treated SOD1^G93A^ male mice (Fig. 5a: median asymptomatic survival: 189 d for control and 174 d for ADAMTS-4 SOD1^G93A^, *P* = 0.0488). Accordingly, there was a significant decrease of the age at symptom onset in ADAMTS-4-treated SOD1^G93A^ male mice (Fig. 5b: average age of onset: 197.8 ± 8.5 d for control and 176.2 ± 3.9 d for ADAMTS-4 SOD1^G93A^, *P* = 0.0423). No change in the latency to fall was observed during the first week following the symptom onset between ADAMTS-4-treated and saline-treated SOD1^G93A^ male mice (Fig. 5c: time latency to fall: 156.7 ± 14.0 s for control and 160.4 ± 5.2 s for ADAMTS-4 SOD1^G93A^, *P* = 0.7573). Surprisingly, ADAMTS-4 treatment did not affect the probability of symptom onset (Fig. 5d: median asymptomatic survival: 241 d for control and 240.5 d for ADAMTS-4 SOD1^G93A^, *P* = 0.4787) or the age at symptom onset in ADAMTS-4-treated SOD1^G93A^ female mice compared to untreated mice (Fig. 5e: average age of onset: 236.0 ± 5.0 d for control and 240.3 ± 6.2 d for ADAMTS-4 SOD1^G93A^, *P* = 0.3798). However, the latency to fall during the first week following the symptom onset was significantly reduced in ADAMTS-4-treated SOD1^G93A^ female mice compared to untreated mice (Fig. 5f: time latency to fall: 157.6 ± 5.3 s for control and 107.8 ± 18.5 s for ADAMTS-4 SOD1^G93A^, *P* = 0.0455). Failure to gain body weight is another indicator of disease onset and progression in SOD1^G93A^ mice, therefore the weight of WT and SOD1^G93A^ mice was recorded from 150 to 190 days for males (Fig. 5g) and from 200 to 240 days for females (Fig. 5h). While no genotype effect was evident in male mice at any time point, there was a genotype effect in female mice. Contrary to WT female mice, the SOD1^G93A^ female mice failed to gain weight over time. We did not observe any change in the weight of ADAMTS-4-treated WT mice (Figs. 5g-h: *P* > 0.05). Overall, our data show that preventing the loss of endogenous ADAMTS-4 activity by exogenous provision of an active human recombinant protein is detrimental for functional outcome in the context of ALS.

### ADAMTS-4 treatment accelerates neurodegeneration in the ventral horn of the lumbar spinal cord in SOD1^G93A^ mice

**Fig. 6:**
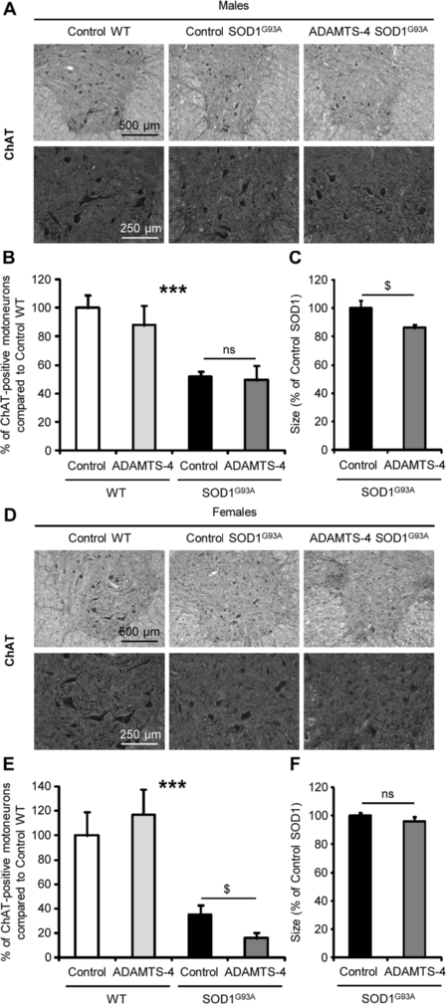
rADAMTS-4 accelerates neurodegeneration in the lumbar spinal cord of SOD1^G93A^ mice. **a** Representative photomicrographs of ventral horns in lumbar spinal cord sections from WT and control or ADAMTS-4-treated SOD1^G93A^ male mice stained with ChAT. Scale bar: 500 or 250 μm. **b-c** Quantification of average spinal motoneuron number (**b**) and size (**c**) in male mice from (**a**). Values plotted are mean ± SEM. Two-way ANOVA: ^***^ *P <* 0.001 WT *Vs* SOD1^G93A^. Unpaired two-tailed t-Test: *P >* 0.05 (Number) or ^$^ *P <* 0.05 (Size) Control *Vs* ADAMTS-4 SOD1^G93A^, *N* = 3 Control WT, *N* = 5 ADAMTS-4 WT, *N* = 8 Control SOD1^G93A^, *N* = 7 ADAMTS-4 SOD1^G93A^. **d** Representative photomicrographs of ventral horns in lumbar spinal cord sections from WT and control or ADAMTS-4-treated SOD1^G93A^ female mice stained with ChAT. Scale bar: 500 or 250 μm. **e-f** Quantification of average spinal motoneuron number (**e**) and size (**f**) in female mice from (**d**). Values plotted are mean ± SEM. Two-way ANOVA: ^***^ *P <* 0.001 WT *Vs* SOD1^G93A^. Unpaired two-tailed t-Test: ^$^ *P <* 0.05 (Number) or *P >* 0.05 (Size) Control *Vs* ADAMTS-4 SOD1^G93A^, *N* = 5 Control WT, *N* = 3 ADAMTS-4 WT, *N* = 5 Control SOD1^G93A^, *N* = 6 ADAMTS-4 SOD1^G93A^

Motoneuron survival in the ventral horn of lumbar spinal cord of WT and SOD1^G93A^ mice was quantified to determine whether the decline of motor function in ADAMTS-4-treated SOD1^G93A^ mice was a result of accelerated spinal cord pathology. In SOD1^G93A^ mice, there was an approximately 50 % loss of motoneurons compared to age-matched WT mice (Fig. 6: *P* < 0.001). ADAMTS-4 did not affect the severity of motoneuron loss in SOD1^G93A^ male mice (Fig. 6a, b: *P* = 0.8297). However, the size of the remaining motoneurons was significantly smaller in ADAMTS-4-treated SOD1^G93A^mice (Fig. 6a, c: −13.8 % for ADAMTS-4-treated SOD1^G93A^ mice compared to untreated SOD1^G93A^ mice, *P* = 0.0496). On the contrary, the number of motoneurons was decreased by approximately 2-fold in ADAMTS-4-treated SOD1^G93A^ female mice compared to untreated SOD1^G93A^ female mice (Fig. 6d, e: −55.7 % for ADAMTS-4-treated SOD1^G93A^ mice compared to untreated SOD1^G93A^ mice, *P* = 0.0347). However, the size of the remaining motoneurons was not different from those of untreated SOD1^G93A^ female mice (Fig. 6d, f: *P* = 0.6961). ADAMTS-4 treatment had no effect on motoneurons in WT mice. Overall, the results demonstrate that ADAMTS-4 promotes neurodegeneration in ALS pathology.

### ADAMTS-4 treatment reduces perineuronal nets enwrapping motoneurons in the ventral horn of the lumbar spinal cord in SOD1^G93A^ mice

**Fig. 7:**
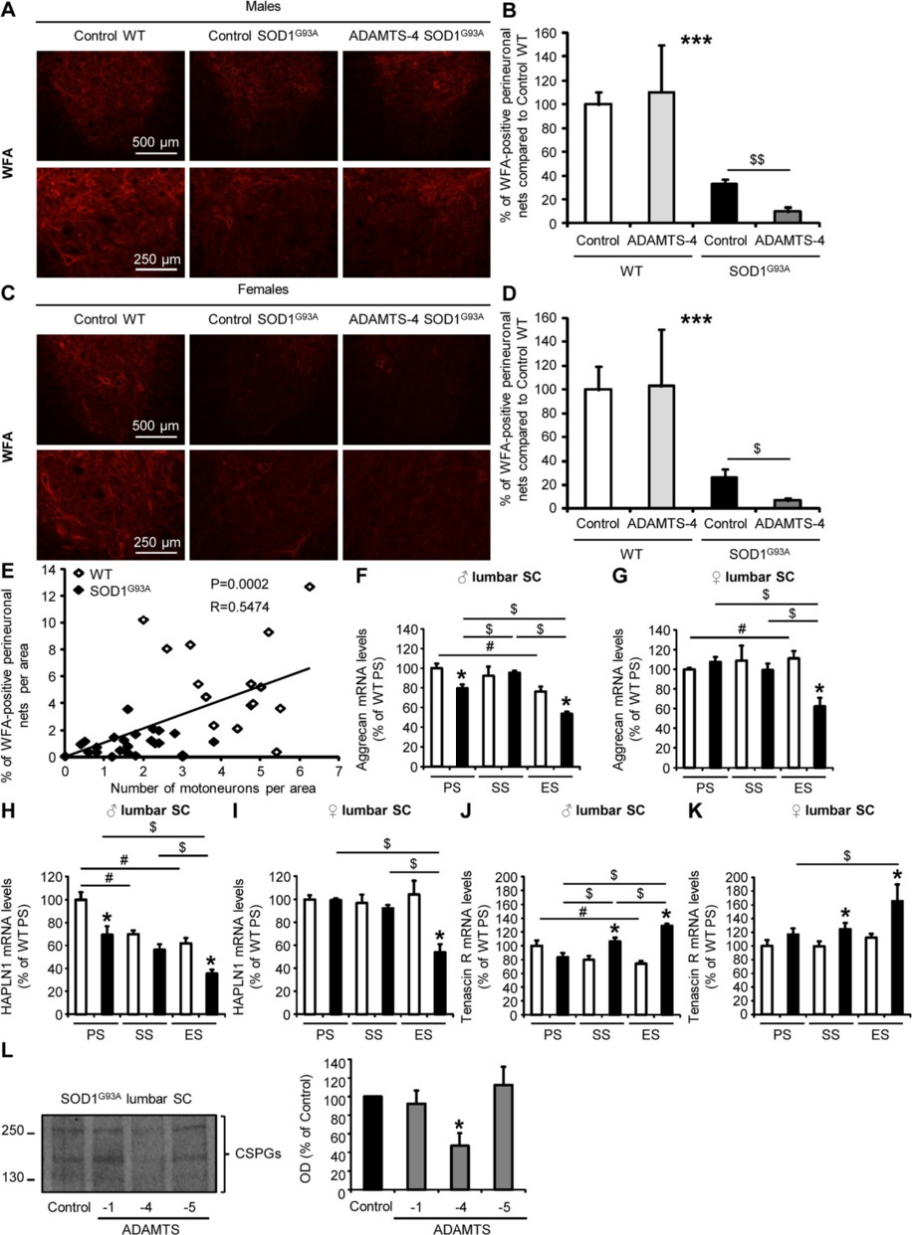
rADAMTS-4 reduces perineuronal nets enwrapping motoneurons in the lumbar spinal cord of SOD1^G93A^ mice. **a** Representative photomicrographs of ventral horns in lumbar spinal cord sections from WT and control or ADAMTS-4-treated SOD1^G93A^ male mice stained with WFA, a marker of perineuronal nets. Scale bar: 500 or 250 μm. **b** Quantification of WFA immunoreactivity per area from male mice (**a**). Values plotted are mean ± SEM. Two-way ANOVA: ^***^ *P <* 0.001 WT *Vs* SOD1^G93A^. Unpaired two-tailed t-Test: ^$$^ *P <* 0.01 Control *Vs* ADAMTS-4 SOD1^G93A^, *N* = 3 Control WT, *N* = 5 ADAMTS-4 WT, *N* = 8 Control SOD1^G93A^, *N* = 7 ADAMTS-4 SOD1^G93A^. **c** Representative photomicrographs of ventral horns in lumbar spinal cord sections from WT and control or ADAMTS-4-treated SOD1^G93A^ female mice stained with WFA. Scale bar: 500 or 250 μm. **d** Quantification of WFA immunoreactivity per area from female mice (**c**). Values plotted are mean ± SEM. Two-way ANOVA: ^***^ *P <* 0.001 WT *Vs* SOD1^G93A^. Unpaired two-tailed t-Test: ^$^ *P <* 0.05 Control *Vs* ADAMTS-4 SOD1^G93A^, *N* = 5 Control WT, *N* = 3 ADAMTS-4 WT, *N* = 5 Control SOD1^G93A^, *N* = 6 ADAMTS-4 SOD1^G93A^. **e** Positive correlation between the percentage of WFA-positive perineuronal nets per area and the number of motoneurons in male and female WT (*N* = 15; blank diamond) and SOD1^G93A^ (*N* = 24; black diamond) symptomatic mice from figs. 4–5. Spearman’s rank correlation: ^***^ *P <* 0.001. R represents the coefficient of correlation. **f-k** Quantitative RT-PCR for aggrecan (**f-g**) HAPLN1 (**h-i**) and tenascin R (**j-k**) in the lumbar spinal cord of WT and SOD1^G93A^ male (**f**, **h**, **j**) and female (**g**, **i**, **k**) mice at PS, SS and ES. Values plotted are mean ± SEM. Mann–Whitney *U*-tests: ^*^ *P <* 0.05 compared to corresponding WT mice, ^$^ *P <* 0.05 compared to SOD1^G93A^ mice at other stages, ^#^ *P <* 0.05 compared to WT mice at other stages, *N* = 3-4. **l** Immunoblot for CSPG (chondroitin sulfate proteoglycans) core proteins in lumbar spinal cord protein extracts of SOD1^G93A^ mice exposed to human recombinant ADAMTS-1, ADAMTS-4 or ADAMTS-5 *ex vivo* for 24 h at 37 °C. Quantification of total CSPG core proteins. Values plotted are mean ± SEM. Mann–Whitney *U*-tests: ^*^ *P <* 0.05 between control and ADAMTS-4 conditions, *N* = 4

To further understand how ADAMTS-4 promotes degeneration/cell death of motoneurons, we then quantified perineuronal nets (PNNs) enwrapping motoneurons, which are protective extracellular matrix (ECM)-envelopes containing chondroitin sulfate proteoglycans (CSPGs), well-known substrates for ADAMTS-4 [17]. For this purpose, lumbar spinal cord sections of SOD1^G93A^ and age-matched WT mice treated or not with ADAMTS-4 were stained with Wisteria Floribunda Agglutin (WFA), a common marker for PNNs [18, 19]. In SOD1^G93A^ mice, there was an approximately 70 % loss of PNNs compared to age-matched WT mice at symptomatic stage (Fig. 7: *P* < 0.001). The amount of the remaining PNNs was even smaller in ADAMTS-4-treated SOD1^G93A^ male (Fig. 7a-b: −69.4 % for ADAMTS-4-treated SOD1^G93A^ mice compared to untreated SOD1^G93A^ mice, *P* = 0.0040) and female (Fig. 7c-d: −74 % for ADAMTS-4-treated SOD1^G93A^ mice compared to untreated SOD1^G93A^ mice, *P* = 0.0210) mice. Additionally, the amount of PNNs was positively correlated with the number of motoneurons in the ventral horn of the spinal cords (Fig. 7e: *P* = 0.0002, R = 0.5474). Again, ADAMTS-4 treatment had no effect on PNNs of WT mice. To determine why the PNNs of SOD1^G93A^ mice were sensitive to ADAMTS-4 treatment while PNNs of WT mice were not, RT-qPCR were carried out for the expression of several PNN components in the lumbar spinal cord of WT and SOD1^G93A^ mice: aggrecan (a CSPG present exclusively in PNNs), HPLAN1 (hyaluronan and proteoglycan link protein 1; involved in the interaction between hyaluronan and CSPGs) and tenascin R (involved in the interaction between the cell surface and CSPGs). Importantly, we observed a decrease of aggrecan expression in the lumbar spinal cord of SOD1^G93A^ male mice compared to WT at the presymptomatic and end-stages of the disease (Fig. 7f: −20.5 % at PS, −30 % at ES compared to age-matched WT, *P* = 0.0339). For female mice, aggrecan mRNA levels were also decreased in the lumbar spinal cord of SOD1^G93A^ mice compared to WT at the end-stage of the disease (Fig. 7g: −34 % at ES compared to age-matched WT, *P* = 0.0339). Then, we observed a decrease of HAPLN1 expression in the lumbar spinal cord of SOD1^G93A^ male mice compared to WT at the presymptomatic and end-stages of the disease (Fig. 7h: −30.7 % at PS, −42.7 % at ES compared to age-matched WT, *P* = 0.0339). For female mice, HAPLN1 mRNA levels were also decreased in the lumbar spinal cord of SOD1^G93A^ mice compared to WT at the end-stage of the disease (Fig. 7i: −48.1 % at ES compared to age-matched WT, *P* = 0.0339). Finally, we observed an increase of tenascin R expression in the lumbar spinal cord of SOD1^G93A^ male and female mice compared to WT at the symptomatic and end-stages of the disease (Fig. 7j: +33.6 % at SS, +74.3 % at ES compared to age-matched male WT, *P* = 0.0339; Fig. 7k: +25.3 % at SS, +47.7 % at ES compared to age-matched female WT, *P* = 0.0433 and 0.0339 respectively). To determine whether the degradation of CSPG core protein present in PNNs was specific of ADAMTS-4 recombinant protein, we exposed or not lumbar spinal cord protein extracts from SOD1^G93A^ male mice at symptomatic stage to human recombinant ADAMTS-1, ADAMTS-4 or ADAMTS-5 *ex vivo* for 24 h at 37 °C. Interestingly, we evidenced that only ADAMTS-4 recombinant protein was successful to degrade CSPG core proteins *ex vivo* (Fig. 7l: −53 % of CSPG core proteins in ADAMTS-4-incubated protein extracts compared to the control condition, *P* = 0.0209; P > 0.05 between ADAMTS-1 or ADAMTS-5-incubated protein extracts and the control condition).

### ADAMTS-4 treatment increases astrogliosis in the ventral horn of the lumbar spinal cord of female SOD1^G93A^ mice

**Fig. 8:**
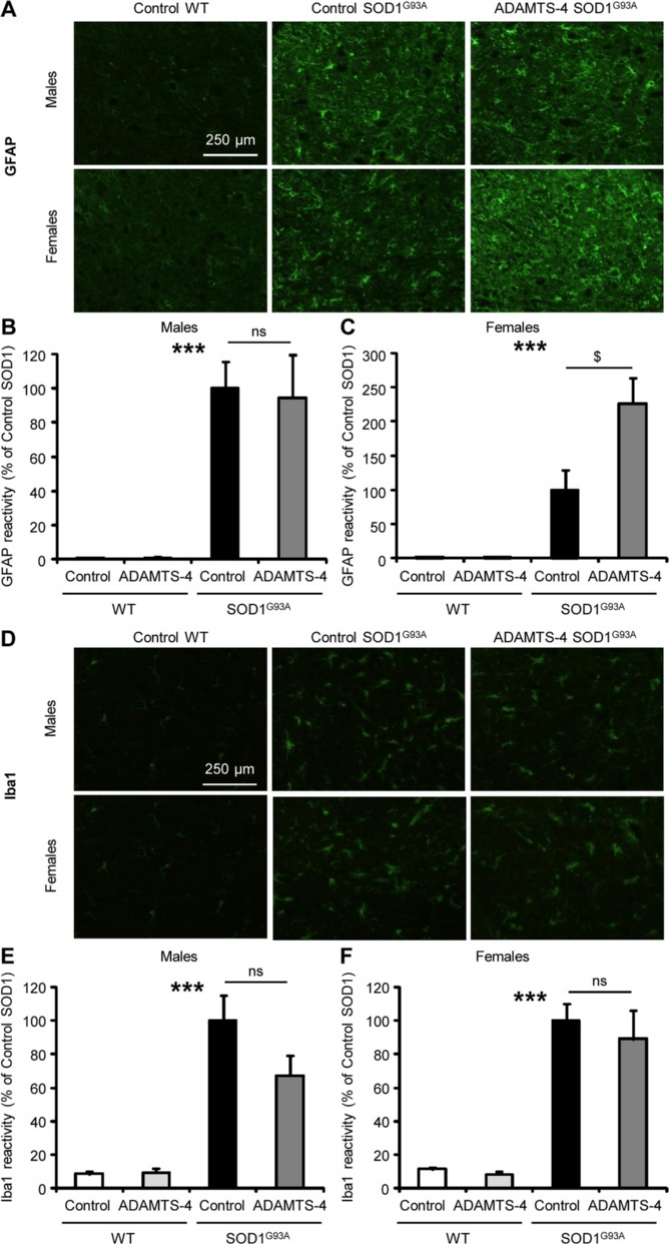
rADAMTS-4 increases astrogliosis in the lumbar spinal cord of female SOD1^G93A^ mice. **a** Representative photomicrographs of ventral horns in lumbar spinal cord sections from WT and control or ADAMTS-4-treated SOD1^G93A^ mice stained with GFAP. Scale bar: 250 μm. **b** Quantification of GFAP immunoreactivity per area from male mice (**a**). Values plotted are mean ± SEM. Two-way ANOVA: ^***^ *P <* 0.001 WT *Vs* SOD1^G93A^. Unpaired two-tailed t-Test: *P >* 0.05 Control *Vs* ADAMTS-4 SOD1^G93A^, *N* = 3 Control WT, *N* = 5 ADAMTS-4 WT, *N* = 8 Control SOD1^G93A^, *N* = 7 ADAMTS-4 SOD1^G93A^. **c** Quantification of GFAP immunoreactivity per area from female mice (**a**). Values plotted are mean ± SEM. Two-way ANOVA: ^***^ *P <* 0.001 WT *Vs* SOD1^G93A^. Unpaired two-tailed t-Test: ^$^ *P <* 0.05 Control *Vs* ADAMTS-4 SOD1^G93A^, *N* = 5 Control WT, *N* = 3 ADAMTS-4 WT, *N* = 5 Control SOD1^G93A^, *N* = 6 ADAMTS-4 SOD1^G93A^. **d** Representative photomicrographs of ventral horns in lumbar spinal cord sections from WT and control or ADAMTS-4-treated SOD1^G93A^ mice stained with Iba1. Scale bar: 250 μm. **e** Quantification of Iba1 immunoreactivity per area from male mice (**d**). Values plotted are mean ± SEM. Two-way ANOVA: ^***^ *P <* 0.001 WT *Vs* SOD1^G93A^. Unpaired two-tailed t-Test: *P >* 0.05 Control *Vs* ADAMTS-4 SOD1^G93A^, *N* = 3 Control WT, *N* = 5 ADAMTS-4 WT, *N* = 8 Control SOD1^G93A^, *N* = 7 ADAMTS-4 SOD1^G93A^. **f** Quantification of Iba1 immunoreactivity per area from female mice (**d**). Values plotted are mean ± SEM. Two-way ANOVA: ^***^ *P <* 0.001 WT *Vs* SOD1^G93A^. Unpaired two-tailed t-Test: *P >* 0.05 Control *Vs* ADAMTS-4 SOD1^G93A^, *N* = 5 Control WT, *N* = 3 ADAMTS-4 WT, *N* = 5 Control SOD1^G93A^, *N* = 6 ADAMTS-4 SOD1^G93A^

Astrogliosis and microgliosis are classical hallmarks of ALS pathology and strongly contribute to neurodegeneration. Therefore, we next investigated whether ADAMTS-4 increases gliosis in the ventral horn of the lumbar spinal cord of SOD1^G93A^ compared to untreated SOD1^G93A^ mice. In SOD1^G93A^ mice, there was a remarkable increase of the astrocyte marker, GFAP (glial fibrillary acidic protein), compared to age-matched WT mice (Fig. 8a-c: *P* < 0.001). While no modification of GFAP expression was observed in ADAMTS-4-treated SOD1^G93A^ male mice compared to untreated SOD1^G93A^ male mice (Fig. 8b: *P* = 0.8514), ADAMTS-4 significantly increased GFAP immunoreactivity in SOD1^G93A^ female mice (Fig. 8c: +126.7 % for ADAMTS-4-treated SOD1^G93A^ mice compared to untreated SOD1^G93A^ mice, *P* = 0.0414). In SOD1^G93A^ mice, there was an approximately 10-fold increase of the microglial marker, Iba1 (ionized calcium-binding adapter molecule-1), compared to age-matched WT mice (Fig. 8d-f: *P* < 0.001). No change in Iba1 expression was observed in ADAMTS-4-treated SOD1^G93A^ mice compared to untreated SOD1^G93A^ mice (Fig. 8e-f: *P* = 0.1146 for males and *P* = 0.5893 for females). GFAP and Iba1 immunoreactivities in WT mice were not altered by ADAMTS-4 treatment.

### ADAMTS-4 treatment does not directly affect neuronal survival in vitro

**Fig. 9:**
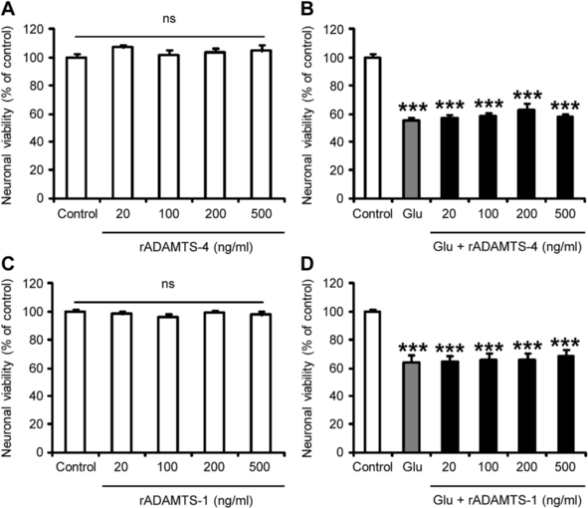
rADAMTS-4 is not toxic to cortical neurons *in vitro*. Neuronal viability assessed by MTT assay in primary cortical neuron cultures treated or not with a human recombinant ADAMTS-4 (**a**-**b**) or ADAMTS-1 (**c**-**d**) at different doses (20, 100, 200, 500 ng/ml) 30 min before exposure (**b**, **d**) or not (**a**, **c**) to glutamate 400 μM (Glu) during 24 h. Values plotted are mean ± SEM. Mann–Whitney *U*-tests: P > 0.05 control *Vs* ADAMTS, ^***^ *P <* 0.001 control *Vs* glutamate, P > 0.05 glutamate *Vs* glutamate + ADAMTS, *N* = 11-12 from 3 independent experiments

Glutamate-induced excitotoxicity represents a key pathophysiological process in ALS contributing to neurodegeneration through activation of Ca^2+^-dependent enzymatic pathways [14]. We therefore aimed at determining whether ADAMTS-4 directly influenced neuronal death in vitro using cortical neurons exposed or not to glutamate for 24 h. Exogenous ADAMTS-4 was not toxic to neurons in control conditions (Fig. 9a: *P* = 0.4158). When neurons were exposed to glutamate, the amount of viable cells was decreased by about 40 % (Fig. 9b, d: *P* < 0.0001). However, ADAMTS-4 did not promote glutamate-induced toxicity (Fig. 9b: *P* = 0.3559, *P* = 0.1962, *P* = 0.2505, *P* = 0.2423 for cell viability when neurons were exposed to glutamate compared to glutamate in presence of ADAMTS-4 at 20, 100, 200, 500 ng/ml respectively). Similarly, we did not observe any influence of exogenous ADAMTS-1 on neuronal viability in control conditions (Fig. 9c: *P* = 0.5896) or after glutamate exposure (Fig. 9d: *P* > 0.9999, *P* = 0.7782, *P* = 0.8880, *P* = 0.5732 for cell viability when neurons were exposed to glutamate compared to glutamate in presence of ADAMTS-1 at 20, 100, 200, 500 ng/ml respectively).

### Exogenous and endogenous ADAMTS-4 modulate the expression and release of neurotrophic factors by glial cells in vitro

**Fig. 10:**
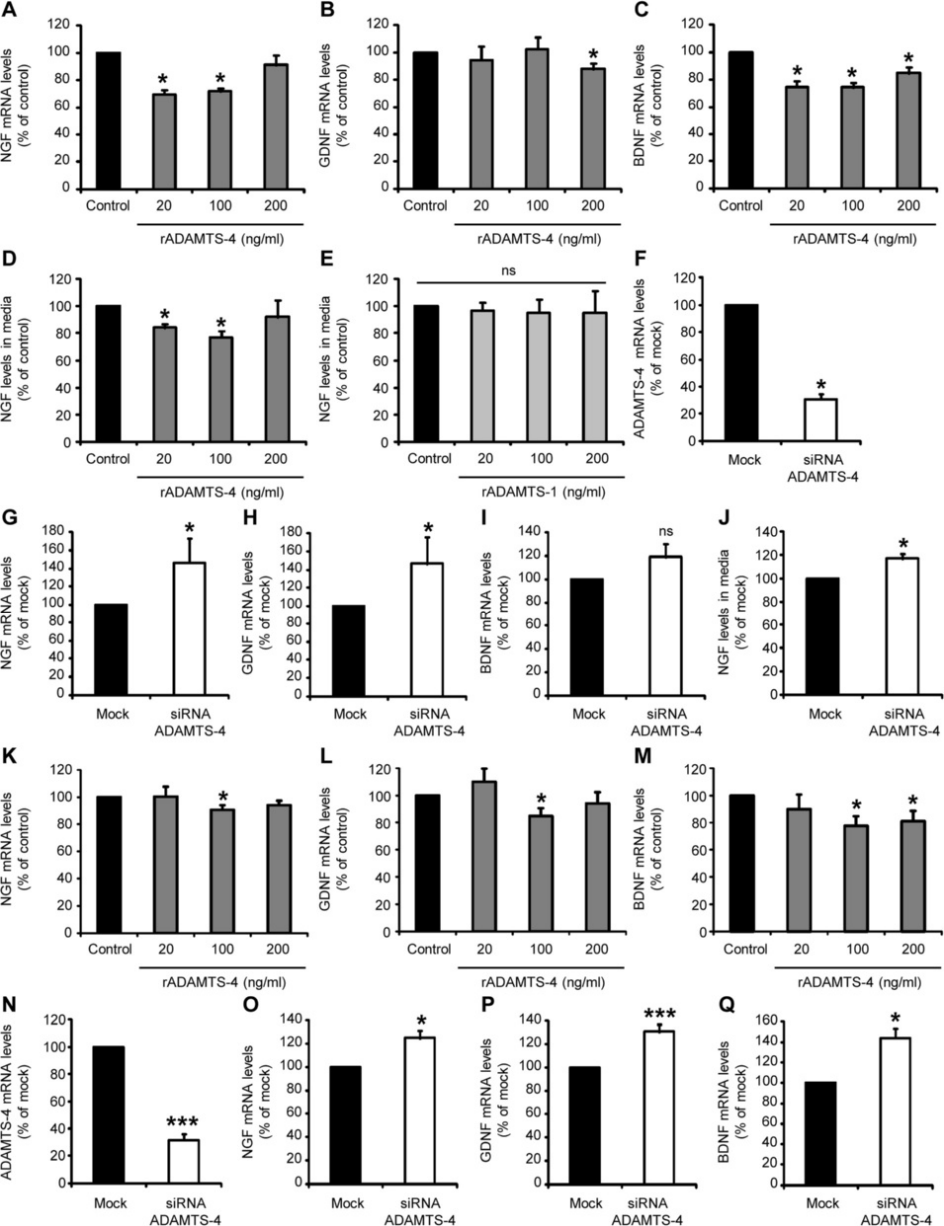
ADAMTS-4 modulates the synthesis/release of neurotrophic factors by glial cells *in vitro*. **a**-**c** Quantitative RT-PCR for NGF (**a**) GDNF (**b**) and BDNF (**c**) expression in mouse adult cortical astrocyte cultures treated or not for 48 h with a human recombinant ADAMTS-4 (20, 100, 200 ng/ml). Values plotted are mean ± SEM. Mann–Whitney *U*-tests: ^*^ *P* < 0.05 compared to control, *N* = 4. **d**-**e** ELISA-measurements of NGF released in the media of mouse adult cortical astrocyte cultures treated or not for 48 h with a human recombinant ADAMTS-4 (**d**) or ADAMTS-1 (**e**) at different doses (20, 100, 200 ng/ml). Values plotted are mean ± SEM. Mann–Whitney *U*-tests: ^*^ *P* < 0.05 (ADAMTS-4) or P > 0.05 (ADAMTS-1) compared to control, *N* = 3. **f**-**i** Quantitative RT-PCR for ADAMTS-4 (**f**) NGF (**g**) GDNF (**h**) and BDNF (**i**) expression in mouse adult cortical astrocyte cultures transfected or not for 48 h with empty vector (mock) or silencing RNAs (siRNAs) targeting the expression of ADAMTS-4. Values plotted are mean ± SEM. Mann–Whitney *U*-tests: ^*^ *P <* 0.05 (ADAMTS-4, NGF, GDNF) or *P >* 0.05 (BDNF) compared to mock, *N* = 4. **j** ELISA-measurements of NGF released in the media of mouse adult cortical astrocyte cultures transfected or not for 48 h with mock or siRNAs targeting the expression of ADAMTS-4. Values plotted are mean ± SEM. Mann–Whitney *U*-tests: ^*^ *P <* 0.05 compared to control, *N* = 4. **k**-**m** Quantitative RT-PCR for NGF (**k**) GDNF (**l**) and BDNF (**m**) expression in mouse neonatal cerebral microglia cultures treated or not for 48 h with a human recombinant ADAMTS-4 (20, 100, 200 ng/ml). Values plotted are mean ± SEM. Mann–Whitney *U*-tests: ^*^ *P <* 0.05 compared to control, *N* = 4. **n**-**q** Quantitative RT-PCR for ADAMTS-4 (**n**) NGF (**o**) GDNF (**p**) and BDNF (**q**) expression in mouse neonatal cerebral microglia cultures transfected or not for 48 h with mock or siRNAs targeting the expression of ADAMTS-4. Values plotted are mean ± SEM. Mann–Whitney *U*-tests: ^*^ *P <* 0.05 (NGF, BDNF), ^***^ *P <* 0.001 (ADAMTS-4, GDNF) compared to mock, *N* = 8

Since ADAMTS-4 did not influence directly neuronal survival in vitro (Fig. 9), we aimed at investigating secondary pathways through which ADAMTS-4 may confer the neurodegeneration/death observed in vivo (Fig. 6). For that purpose, we investigated whether ADAMTS-4 may modulate the expression and/or release of neurotrophic factors such as NGF (nerve growth factor), GDNF (glial cell-derived neurotrophic factor) and BDNF (brain-derived neurotrophic factor), in astrocyte and microglia cultures. ADAMTS-4 was found to decrease the mRNA levels of NGF (Fig. 10a: −30.2 % or −28.1 % for astrocytes treated with ADAMTS-4 at 20 or 100 ng/ml compared to control astrocytes, *P* = 0.0209), GDNF (Fig. 10b: −11.9 % for astrocytes treated with ADAMTS-4 at 200 ng/ml compared to control astrocytes, *P* = 0.0209) and BDNF (Fig. 10c: −25.2 %, −25.6 % or −14.9 % for astrocytes treated with ADAMTS-4 at 20, 100 or 200 ng/ml compared to control astrocytes, *P* = 0.0209) in astrocytes. The reduction of NGF mRNA levels by ADAMTS-4 (Fig. 10a) was accompanied by a decrease of NGF present in the culture media (Fig. 10d: −15.9 % or −23.1 % for astrocytes treated with ADAMTS-4 at 20 or 100 ng/ml compared to control astrocytes, *P* = 0.0495). This effect was specific to ADAMTS-4, since a human recombinant ADAMTS-1 did not change the levels of NGF present in the culture media (Fig. 10e: *P* = 0.5127). ADAMTS-4 was not toxic to cultured astrocytes.

To confirm these results, we transfected astrocytes for 2 h with silencing siRNAs targeting ADAMTS-4 or with an empty vector (mock) as a control. After 48 h, ADAMTS-4 gene expression was decreased by 69 % in astrocytes transfected with siRNAs silencing ADAMTS-4 expression compared to control astrocytes (Fig. 10f: *P* = 0.0209). Interestingly, we observed an increase of the mRNA levels of NGF (Fig. 10g: +46.1 % for astrocytes transfected with siRNAs targeting ADAMTS-4 compared to control astrocytes, *P* = 0.0209) and GDNF (Fig. 10h: +46.8 % for astrocytes transfected with siRNAs targeting ADAMTS-4 compared to control astrocytes, *P* = 0.0209), but not BDNF (Fig. 10i: *P* = 0.2482), in astrocytes transfected with siRNAs silencing ADAMTS-4 expression compared to control astrocytes. In the culture media, we observed an increase of NGF in astrocytes transfected with siRNAs silencing ADAMTS-4 expression compared to control astrocytes (Fig. 10j: +16.9 % for astrocytes transfected with siRNAs targeting ADAMTS-4 compared to control astrocytes, *P* = 0.0209).

To determine whether this effect was astrocyte-specific, we repeated the same experiments in microglia cultures. ADAMTS-4 decreased the mRNA levels of NGF (Fig. 10k: −9.4 % for microglia treated with ADAMTS-4 at 100 ng/ml compared to control microglia, *P* = 0.0209), GDNF (Fig. 10l: −15.1 % for microglia treated with ADAMTS-4 at 100 ng/ml compared to control microglia, *P* = 0.0209) and BDNF (Fig. 10m: −22.4 % or −18.9 % for microglia treated with ADAMTS-4 at 100 or 200 ng/ml compared to control microglia, *P* = 0.0209) in microglia cultures. ELISA failed to detect NGF in microglia culture media (data not shown). ADAMTS-4 was not toxic to cultured microglia.

To confirm these results, we transfected microglial cells for 2 h with silencing siRNAs targeting ADAMTS-4 or with an empty vector (mock) as a control. After 48 h, ADAMTS-4 gene expression was decreased by 68 % in microglial cells transfected with siRNAs silencing ADAMTS-4 expression compared to control microglia (Fig. 10n: *P* = 0.0008). Importantly, we observed an increase in the mRNA levels of NGF (Fig. 10o: +25.1 % for microglia transfected with siRNAs targeting ADAMTS-4 compared to control microglia, *P* = 0.0117), GDNF (Fig. 10p: +30.7 % for microglia transfected with siRNAs targeting ADAMTS-4 compared to control microglia, *P* = 0.0008) and BDNF (Fig. 10q: +44.2 % for microglia transfected with siRNAs targeting ADAMTS-4 compared to control microglia, *P* = 0.0117) in microglial cells transfected with siRNAs silencing ADAMTS-4 expression compared to control microglia.

### ADAMTS-4 treatment decreases NGF expression in the ventral horn of the lumbar spinal cord of male SOD1^G93A^ mice

**Fig. 11:**
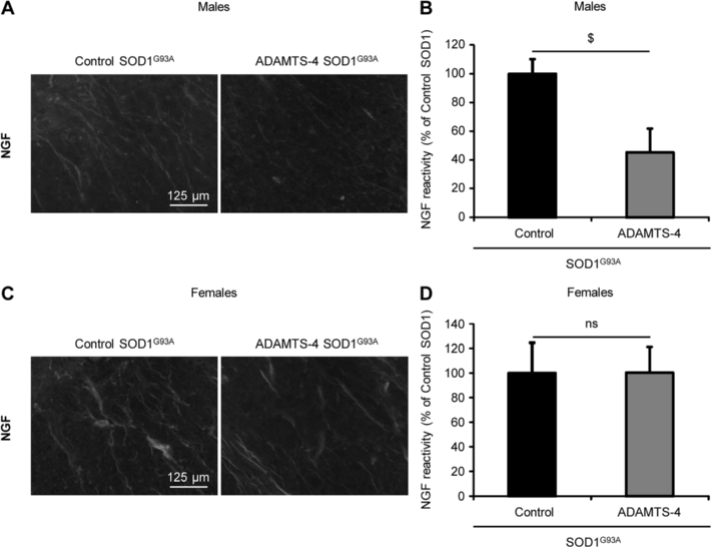
rADAMTS-4 decreases NGF expression in the lumbar spinal cord of male SOD1^G93A^ mice. **a** Representative photomicrographs of ventral horns in lumbar spinal cord sections from control or ADAMTS-4-treated SOD1^G93A^ male mice stained with NGF. Scale bar: 125 μm. **b** Quantification of NGF immunoreactivity per area from male mice (**a**). Values plotted are mean ± SEM. Unpaired two-tailed t-Test: ^$^ *P <* 0.05 Control *Vs* ADAMTS-4 SOD1^G93A^, *N* = 8 Control SOD1^G93A^, *N* = 7 ADAMTS-4 SOD1^G93A^. **c** Representative photomicrographs of ventral horns in lumbar spinal cord sections from control or ADAMTS-4-treated SOD1^G93A^ female mice stained with NGF. Scale bar: 125 μm. **d** Quantification of NGF immunoreactivity per area from female mice (**c**). Values plotted are mean ± SEM. Unpaired two-tailed t-Test: *P >* 0.05 Control *Vs* ADAMTS-4 SOD1^G93A^, *N* = 5 Control SOD1^G93A^, *N* = 6 ADAMTS-4 SOD1^G93A^

We next aimed to determine whether the regulation of neurotrophic factor expression and release by ADAMTS-4 evidenced in vitro (Fig. 10) may contribute at least partly to the deleterious effects of ADAMTS-4 observed in vivo in SOD1^G93A^ mice. For that purpose, we immunostained NGF in lumbar spinal cord sections of SOD1^G93A^ mice treated or not with ADAMTS-4. Interestingly, we observed a 2-fold decrease of NGF expression in the ventral horn of the lumbar spinal cords of SOD1^G93A^ male mice treated with ADAMTS-4 compared to untreated SOD1^G93A^ mice (Fig. 11a-b: −54.9 % for ADAMTS-4-treated SOD1^G93A^ mice compared to untreated SOD1^G93A^ mice, *P* = 0.0209). However, no difference of NGF expression was found between SOD1^G93A^ female mice treated or not with ADAMTS-4 (Fig. 11c-d: *P* = 0.9907).

## Discussion

**Fig. 12:**
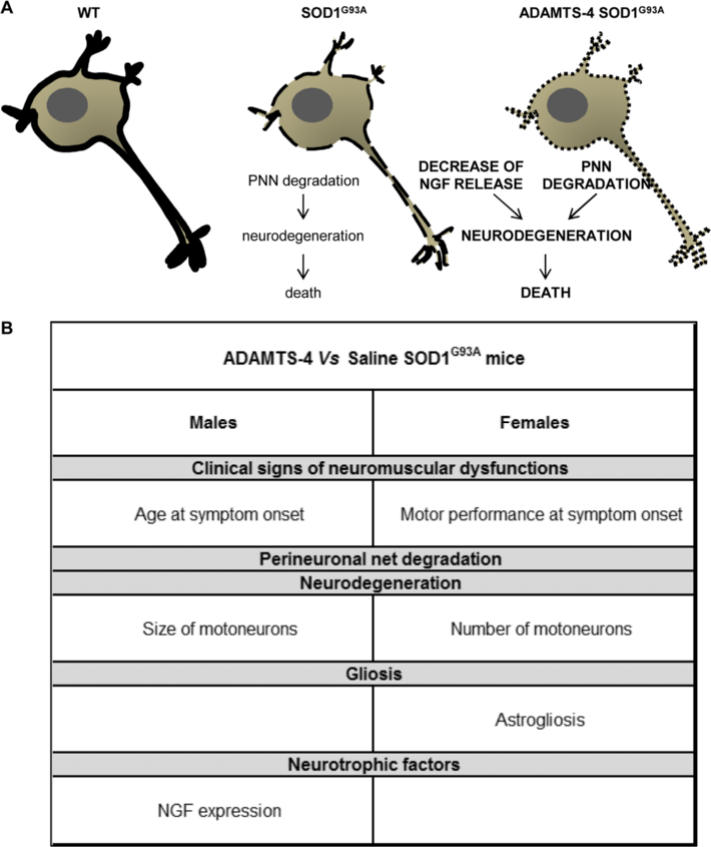
Gender similarities and differences in the effect of ADAMTS-4 treatment on ALS. **a** Schematic representation of ADAMTS-4 treatment promoting the decline of NGF production and ALS-induced perineuronal net degradation which contribute to the degeneration and even death of motoneurons in the ventral horn of the lumbar spinal cord of SOD1^G93A^ mice. **b** A table describing the similarities and differences observed in behavioral and anatomical effects of ADAMTS-4 treatment in SOD1^G93A^ male and female mice

ADAMTS-4 is a metalloproteinase specialized in the degradation of chondroitin sulfate proteoglycans (CSPGs) whose functions during neurodegenerative diseases, including ALS, have not been investigated. Here, we demonstrated that (i) ADAMTS-4 activity is decreased at disease end-stage in the spinal cord of SOD1^G93A^ mice, and that (ii) provision of exogenous ADAMTS-4 promoted the degradation of perineuronal nets (PNNs) and decreased glial production of neurotrophic factors, possibly thereby enhancing neurodegeneration and subsequent motor impairments in SOD1^G93A^ mice (Fig. 12a).

In contrast to other ADAMTS proteoglycanases, ADAMTS-4 is highly expressed in the CNS in all types of cells [1, 20–23]. However, ADAMTS proteoglycanases display different potency for CSPGs. For example, ADAMTS-5 is more potent than ADAMTS-4 for the proteolysis of aggrecan [24, 25], a CSPG found exclusively in PNNs. Nevertheless, ADAMTS functions extend beyond proteolysis by regulating synaptic protein expression (ADAMTS-1) [3] or neurotrophic factor expression/release (ADAMTS-4). Modifications of ADAMTS-4 expression/activity have been reported during spinal cord injury (SCI), experimental autoimmune encephalomyelitis and multiple sclerosis [1]. Here, we showed that ADAMTS-4 activity was decreased in the lumbar spinal cord of SOD1^G93A^ mice compared to WT mice at disease end-stage. This reduction could be related to a myriad of events, including a decrease of ADAMTS-4 gene/protein expression, an increase of the proteolytic cleavage of ADAMTS-4 mature form, and an increase of its inhibitor TIMP-3. TIMP-3 is also an inhibitor for ADAMTS-1 and −5, which may impair their activities although their expressions are increased in the lumbar spinal cord of SOD1^G93A^ mice.

We and others have previously shown that exogenous supply of ADAMTS-4 after SCI in rats promotes neuroplasticity by degrading CSPGs and subsequent functional recovery [5, 6]. One possible reason why ADAMTS-4 is beneficial after SCI while it is deleterious during ALS may rely on the impact of CSPGs/PNNs degradation in these two diseases. Indeed, while it is clear that CSPGs are highly induced after SCI and represent strong inhibitors for neuroregeneration in this context, their expression and role during neurodegenerative diseases including ALS are poorly understood [18, 26–28]. Nevertheless, increasing evidence shows that the neurons devoid of a net are less protected against neurodegeneration compared to PNNs-bearing neurons in Alzheimer disease (AD) or oxidative stress animal models [29–31]. Forostyak and colleagues have shown that the PNNs enwrapping spinal motoneurons of SOD1^G93A^ rats are considerably degraded at disease end-stage compared to WT rats. Additionally, they showed that PNNs are partly preserved in SOD1^G93A^ rats after transplantation of bone marrow mesenchymal stromal cells and that this effect is associated with an increase of motoneuron survival and an increase of SOD1^G93A^ rats survival [18]. Similarly, we showed here a decrease of PNNs around motoneurons of the lumbar spinal cord of SOD1^G93A^ mice at the symptomatic stage. Disorganized SOD1^G93A^-PNNs may facilitate local degradation of the remaining aggrecan by ADAMTS-4 since PNNs were even more damaged in ADAMTS-4-treated SOD1^G93A^ mice. This was associated with an increased neurodegeneration and a poor functional outcome. Our results suggest that digestion of PNNs by ADAMTS-4 may be harmful for motoneurons during ALS pathology. Because PNNs only contain 2 % of total CSPGs [32], we cannot exclude that ADAMTS-4 may have an effect on digestion of the 98 % remaining CSPGs.

Neurotrophic factors have been extensively described to protect dying motoneurons and represent a potential therapeutic strategy in ALS [33, 34]. Here we described for the first time that ADAMTS-4 decreased the expression of several neurotrophic factors in astrocytes and microglia. Accordingly, decreasing ADAMTS-4 expression by siRNA approach led to an increase of neurotrophic factor expression. This demonstrates that ADAMTS-4 functions are not limited only to CSPGs degradation. It would be interesting to determine whether ADAMTS-4 may induce mechanisms previously described to downregulate neurotrophic factor production, for instance, by modulating the nuclear translocation of transcription factors such as the histone deacetylase HDAC6 (negative regulator) [35], CREB (cAMP response element-binding protein) or NF-κB (nuclear factor kappa B) (positive regulators) [36–38], and/or by modulating micro-RNAs (miR) production such as miR-15a, miR-132, miR-134, miR-221 or Let-7 miR [39–41]. Because ADAMTS-4 did not increase glutamate-induced neuronal death in vitro, we hypothesize that ADAMTS-4-dependent decrease of neurotrophic factors released by glial cells around dying motoneurons during ALS may participate in the accelerated neurodegeneration induced by ADAMTS-4 in vivo. The neurotrophic factor production after ADAMTS-4 treatment has not been studied in SCI studies so far, but we could hypothesize that even if ADAMTS-4 also induced a decrease of neurotrophic factor production in the context of SCI, its impact may be negligible compared to the great benefit of the CSPGs/PNNs degradation-induced neuroregeneration [5, 6]. Among the neurotrophic factors modulated by ADAMTS-4, NGF is of particular interest as it exerts dual roles on neuronal survival/cell death depending on whether it activates the tyrosine kinase receptor TrkA or the tumor necrosis factor receptor p75^NTR^ [42], two receptors induced after injury and in ALS [43, 44]. Although astrocyte-derived NGF was described to promote motoneuron cell death through the activation of p75^NTR^ receptor during ALS [45, 46], it was also described that the surviving motoneurons expressed the TrkA receptor [43], suggesting that NGF-TrkA signaling also plays a critical role in the survival of motoneurons. Additionally, NGF-p75^NTR^ signaling reduces astrocyte proliferation in vitro and in vivo in an autocrine manner [47]. ADAMTS-4-mediated reduction in NGF release by astrocytes may contribute to neurodegeneration in ADAMTS-4-treated SOD1^G93A^ mice by preventing TrkA signaling in surviving motoneurons. The decrease of astrocytic NGF-p75^NTR^ signaling could also explain the increase in astrocyte activation/proliferation observed in ADAMTS-4-treated SOD1^G93A^ females. Contrary to ADAMTS-4-treated SOD1^G93A^ males, no modification of NGF expression was identified in the lumbar spinal cords of ADAMTS-4-treated SOD1^G93A^ females. Nevertheless, we cannot rule out that: i) the decrease of NGF in ADAMTS-4-treated SOD1^G93A^ females may have occurred earlier than the time point studied here. ii) the main source of NGF is provided by astrocytes, therefore the increased astrogliosis observed in the lumbar spinal cord of ADAMTS-4-treated SOD1^G93A^ females might mask the reducing effect of ADAMTS-4 on NGF astrocytic expression.

This study reveals intriguing gender-specific effects of ADAMTS-4 at the functional and anatomical levels (Fig. 12b). While ADAMTS-4-treated SOD1^G93A^ males presented clinical signs of neuromuscular dysfunctions 20 days earlier than untreated SOD1^G93A^ males, ADAMTS-4-treated SOD1^G93A^ females had symptoms of neuromuscular dysfunctions at the same age as untreated SOD1^G93A^ females. Even though we evidenced that the motor performance of ADAMTS-4-treated SOD1^G93A^ females was more impaired than untreated SOD1^G93A^ females at symptom onset, it is clear that ADAMTS-4 more severely impaired the functional outcome of SOD1^G93A^ males than females. Surprisingly, this does not reflect what happened at the anatomical level, since ADAMTS-4 caused motoneuron death in SOD1^G93A^ females and only mild motoneuron degeneration in SOD1^G93A^ males. It is difficult to explain such non-linear relationship between functional and anatomical outcomes in ADAMTS-4-treated SOD1^G93A^ males (severe functional impairment/mild neurodegeneration) or females (mild functional impairment/severe neurodegeneration). However, the fact that motoneuron degeneration obviously led to cell death in ADAMTS-4-treated SOD1^G93A^ females but not yet in males may be due to the increased astrogliosis induced by ADAMTS-4 in SOD1^G93A^ females, but not in males. Estrogens may most likely play a role in some of the mechanisms mediated by ADAMTS-4. Gender-specific effects of another ADAMTS proteoglycanase, ADAMTS-1, have been previously observed in the CNS where a decline of synaptic proteins was evidenced in the frontal cortex of ADAMTS-1 knock-out female mice during development, but not in males [3].

## Conclusions

To conclude, our results provide the first evidence that ADAMTS-4 promotes neurodegeneration in the context of ALS. It would be interesting to determine if: (i) endogenous ADAMTS-4 contributes to neurodegeneration in mice expressing high copy number of mutant SOD1 as well as in other model of ALS such as WT or mutant TDP43 (TAR DNA-binding protein 43) rodent models, or even in frontotemporal dementia or AD models; (ii) therapeutic approaches aimed at decreasing ADAMTS-4 expression/activity would represent potential targets to slow down neurodegeneration in chronic CNS diseases.

## Methods

### Ethics

Animal experiments were conducted according to the national regulation of the usage and welfare of laboratory animals, approved by the National Animal Experiment Board of Finland and followed the Council of Europe legislation and regulation for animal protection.

### Animals

Transgenic male and female mice over-expressing the human superoxide dismutase SOD1^G93A^ mutation were also purchased from the Jackson laboratory (Bar Harbor, Maine, USA) and maintained on C57BL/6 J congenic background. Transgenic genotypes were identified by polymerase chain reaction (PCR) amplification of ear DNA a few days after birth and of liver DNA after death to confirm the results of the first genotyping. PCR revealed a low copy number of mutated SOD1 in the mice used in this study. The mice were housed under controlled temperature, humidity and light conditions (12 h light and dark cycles) with free access to food and water. Animals of the same sex were housed in groups of up to 5 in cages. WT and SOD1^G93A^ mice were used for the 2 studies described hereafter.

### Study 1: ADAMTS-4 expression in the time course of ALS

- *Characterization of the disease stage in SOD1*^*G93A*^*mice.* Male and female SOD1^G93A^ mice and WT littermates from generations 18–19 were sacrificed at key time points during the development of the ALS pathology: presymptomatic (m/f: ~13/14.5 weeks-old), symptomatic (m/f: ~20.5/22 weeks-old) and end-stages (m/f: ~27/28 weeks-old). The symptomatic stage was estimated based on the symptom onset of SOD1^G93A^ mice from the previous cohort, and confirmed when the mice developed abnormal hindlimb splay reflexes when suspended by their tails. The end-stage was defined as the age when the mice suffered from functional paralysis of the hindlimbs. At disease end-stage, SOD1^G93A^ mice were given macerated food for easier food uptake and hydration. Mice were sacrificed by terminal perfusion with heparinized saline, followed or not by paraformaldehyde (PFA) perfusion (as described in the immunohistochemistry section) for respectively RNA (*N* = 3-4 in each group)/protein (*N* = 4 in each group) (cortices, cervical, thoracic and lumbar spinal cords) or staining (*N* = 3 in each group) (lumbar spinal cords) purposes.

### Study 2: ADAMTS-4 treatment and functional outcome in ALS

- *Intracerebroventricular injection of recombinant ADAMTS-4.* Male and female WT and SOD1^G93A^ mice from generations 20–22 were randomized into treatment groups using GraphPad Quickcalcs (GraphPad Software Inc., La Jolla, CA, USA): *N* = 5 Control WT males, *N* = 5 ADAMTS-4 WT males, *N* = 9 Control SOD1^G93A^ males, *N* = 9 ADAMTS-4 SOD1^G93A^ males, *N* = 5 Control WT females, *N* = 4 ADAMTS-4 WT females, *N* = 7 Control SOD1^G93A^ females, *N* = 6 ADAMTS-4 SOD1^G93A^ females. The anesthesia of mice was induced by 5 % isoflurane in a 70 %/30 % mixture of NO_2_/O_2_ and maintained at 2 % isoflurane during the surgery. The temperature of the mice was controlled by a homeothermic control system connected to a heating blanket and rectal probe (Harvard apparatus, Pan Lab, Barcelona, Spain). A 4-μl volume containing saline or 40 ng of a human recombinant ADAMTS-4 (CC1028, Merck Millipore, Darmstadt, Germany) was injected bilaterally into lateral ventricles using a 5-μl Hamilton syringe (Hamilton company, Reno, Nevada, USA) at the age of 9 and 13 weeks (coordinates from Bregma: mediolateral = ± 1 mm, anteroposterior = − 0.5 mm, dorsoventral = − 3 mm).
- *Assessment of functional outcome.* Disease onset was determined by the wire hang test [48]. Each mouse was placed on a wire lid of a conventional cage which was turned upside down and the latency of the mouse to fall was recorded. Deficits in motor performance were defined by the inability to hang for more than 3 min. If the mouse fell, the test was repeated for the second time. The test was performed 3 times a week. In parallel, the weight of the transgenic mice was recorded 3 times a week using a normal digital balance, while WT mice were only weighted once a week. The testing was performed blinded to the experimental groups. Mice were sacrificed during the symptomatic stage (m/f: ~29/35 weeks-old) by terminal perfusion with heparinized saline, followed by PFA perfusion (as described in the immunohistochemistry section) for staining (lumbar spinal cords) purposes.

### Quantitative real-time PCR

Total RNAs were isolated by homogenizing spinal cords or cortex in TRIzol (Life technologies, Carlsbad, CA, USA) according to manufacturer’s instructions utilizing 5-mm stainless steel beads and a Tissuelyzer II homogenizator (Qiagen, Leusden, NL, USA). Total RNAs from cells were isolated with the RNeasy Mini Kit (Qiagen). Synthesis of cDNA was performed by using 500 ng of total RNA, Maxima reverse transcriptase, dNTP and random hexamer primers (Life technologies). The final concentration of cDNA was 2.5 ng/μl. The relative expression levels of mRNAs encoding the selected genes were run in duplicates and measured according to the manufacturer’s protocol by quantitative RT-PCR (StepOne Plus™ Real-Time PCR system; Life technologies) and using specific assays-on-demand target mixes (Life technologies) as follows: ADAMTS-1: Mm00477355_m1; ADAMTS4: Mm00556068_m1; ADAMTS-5: Mm00478620_m1; ADAMTS-9: Mm00614433_m1; TIMP-3 (type 3 tissue inhibitor of metalloproteinases): Mm00441826_m1; Aggrecan: Mm00545794_m1; BDNF (brain-derived neurotrophic factor): Mm0133402_m1; NGF (nerve growth factor): Mm00443039_m1; GDNF (glial cell-derived neurotrophic factor): Mm00599849_m1; HAPLN1 (hyaluronan and proteoglycan link protein 1): Mm00488952_m1; Tenascin R: Mm00659075_m1; GAPDH (glyceraldehyde-3-phosphate dehydrogenase): 4352932E (Applied Biosystems, Warrington, UK). The expression levels were normalized to GAPDH. Relative mRNA transcription was expressed as a percentage of control conditions using the 2^─∆∆*Ct*^ method where *Ct* is the threshold-cycle value. The relative expression of ADAMTS proteoglycanases was expressed as a percentage of ADAMTS-1 gene expression using the 2^─∆∆*Ct*^ method: 2^-((∆Ct ADAMTS-1 - ∆Ct GAPDH) - (∆Ct ADAMTS-4, 5 or 9 - ∆Ct GAPDH))^.

### Protein extraction

Cortices as well as cervical, thoracic or lumbar spinal cords were dissociated in ice-cold TNT buffer (50 mM Tris–HCl pH 7.4; 150 mM NaCl; 0.5 % Triton X-100) containing EDTA/EGTA (ethylene diamine/glycol tetraacetic acid, 1 mM), protease (Sigma-Aldrich, St Louis, MO, USA) and phosphatase (Roche Diagnostics, Mannheim, Germany) inhibitors. For the ADAMTS-4 fluorimetric assay, lumbar spinal cords were dissociated in TNT buffer without protease and phosphatase inhibitors. Debris were removed by centrifugation (12,000 g at 4 °C, 15 min). Supernatants were stored at −70 °C until further processing. Protein quantification was performed according to the BCA protein method (Pierce, Rockford, USA).

### Western blot

Proteins (5 μg) were resolved on 12 % polyacrylamide gel under denaturing conditions and transferred onto a polyvinylidene difluoride membrane. Membranes were blocked with phosphate buffered saline (PBS) tween (0.2 % Tween-20; Sigma-Aldrich) and 5 % of milk. Blots were incubated overnight at 4 °C with the rabbit anti-ADAMTS-4 (1/5000; AbCam, Cambridge, UK) or the rabbit anti-ADAMTS-5 (1/1000; AbCam) primary antibodies diluted in PBS-tween containing 5 % of bovine albumin serum (BSA). After a 2-h incubation at room temperature (RT) with the peroxidase-conjugated anti-rabbit secondary antibody (1/2000; GE Healthcare life sciences, Uppsala, Sweden), proteins were revealed with an enhanced chemiluminescence ECL-Plus kit immunoblotting detection system (GE Healthcare life sciences) and visualized using Storm^TM^ FluorImager system. Mouse anti-β-actin (1/5000; Sigma-Aldrich) was used as a loading control and visualized by Alexa fluor 647-conjugated anti-mouse secondary antibody (1/1000; Jackson ImmunoResearch laboratories Inc., West Grove, PA, USA).

### Ex vivo CSPG proteolysis by ADAMTS human recombinant proteins

Sixteen μg of lumbar spinal cord protein extracts from symptomatic SOD1^G93A^ male mice were exposed or not to 1 μg of human recombinant ADAMTS-1 (2197-AD-020), ADAMTS-4 (4307-AD-020) or ADAMTS-5 (2198-AD-020; all recombinant proteins were from R&D SYSTEMS, Minneapolis, MN, USA) in the aggrecanase buffer (50 mM TrisHCl, 125 mM NaCl, 5 mM CaCl_2_, pH 7.5) within a final volume of 55 μl, for 24 h at 37 °C (*N* = 4 per condition). The reaction was stopped by heating the samples at 75 °C for 10 min. Twelve μl of each condition of the above preparations were resolved in a 6 % polyacrylamide gel, the membrane probed with the mouse anti-CSPG antibody (1/1000; Sigma-Aldrich), then with the peroxidase-conjugated anti-mouse secondary antibody (1/5000; GE Healthcare life sciences) and finally revealed by ECL detection, as previously described in the Western Blot section.

### Fluorimetric assay for ADAMTS-4

A fluorogenic substrate (5-FAM/TAMRA; SensoLyte® 520 Aggrecanase-1 assay kit, Eurogentec, San Jose, CA) was incubated with protein extracts of lumbar spinal cords (25 μg in 50 μl) of WT and SOD1^G93A^ male and female mice at presymptomatic, symptomatic and end-stages (*N* = 4 in each group). Measurements were performed at 37 °C over 60 min using a multiplate reader. The slope of each absorbance curve was then determined between 10 and 20 min.

### Immunohistochemistry

Anesthetized mice were perfused with cold heparinized saline and, thereafter, with a solution containing 4 % PFA in 0.1 M phosphate buffer (PB) pH 7.4. Lumbar spinal cords were removed and rinsed in a PB containing 20 % sucrose for cryoprotection for 24 h. The spinal cords were embedded and frozen in OCT (Optimal Cutting Temperature; Sakura Finetek, Tokyo, Japan). Five 12-μm-transverse sections 200 μm apart covering a 1-mm-length of each lumbar spinal cord were cut on a cryostat, collected on lysine glasses (Thermo Scientific, UK), and stored at −70 °C until analysis. After washing with PB, PBS and PBS-tween (0.05 % Tween-20), sections were treated as required with PBS-TritonX-100 (0.4 %, Sigma-Aldrich) and unspecific binding was blocked with 1 h incubation with 10 % normal goat or rabbit serum (NGS or NRS; Vector Laboratories Ltd, Burlingame, CA) or 0.5 % mouse on mouse reagent (MOM; Vector Laboratories Ltd). Incubation with primary antibodies was conducted overnight at RT with dilutions as follows: rabbit anti-ADAMTS-4 (1/500; AbCam), rabbit anti-GFAP (glial fibrillary acidic protein, 1/200; Dako, Glostrup, Denmark), mouse anti-GFAP (1/400; Merck Millipore), rabbit anti-Iba1 (ionized calcium-binding adapter molecule-1, 1/250; Wako Pure Chemical Industries, Ltd, Tokyo, Japan), mouse anti-NeuN (1/200; Chemicon, Billerica, MA, USA), rabbit anti-NGF (1/100; AbCam), mouse anti-APC (Adenomatous polyposis coli, 1/200; Merck Millipore) or lectin from Wisteria Floribunda (WFA, Wisteria Floribunda Agglutin, 1/1000; Sigma-Aldrich). After washing with PBS-tween, sections were incubated with corresponding fluorescent Alexa fluor −488 or −568-conjugated secondary antibodies (1/200; Life technologies) or with a fluorescent Alexa fluor-568 secondary antibody conjugated to streptavidin (for WFA staining, 1/500; Life technologies) for 2 h at RT, then washed again and finally mounted in Vectashield with DAPI (Vector Laboratories Ltd). For choline acetyltransferase (ChAT) staining, sections were incubated in 0.3 % H_2_O_2_ diluted in MeOH for 30 min to block endogenous peroxidase activity followed by heat-mediated antigen retrieval for 30 min in 0.05 M citrate buffer, pH 6.0. After blocking unspecific binding, sections were incubated in primary antibody (1/500; Chemicon), followed on the next day by 1-h incubation in biotinylated secondary antibody (1/200) and then incubation in avidin-biotin complex solution (Vectastain Elite kit, both from Vector Laboratories Inc., USA). The staining was visualized using nickel-enhanced diaminobenzidine (Sigma-Aldrich) with 0.075 % H_2_O_2_ as chromogen/substrate reagent solution. Negative controls for unspecific binding of the secondary antibodies were conducted in parallel sections following the same procedures described above except the incubation in primary antibodies.

For GFAP, Iba1, WFA, ChAT and NGF analyses, the ventral horn of the lumbar spinal cords were imaged using 10x (GFAP, Iba1, ChAT), 20x (WFA) or 40x (NGF) magnification on an AX70 microscope (Olympus corporation, Tokyo, Japan) coupled to a digital camera (Color View 12, soft Imaging System, Muenster, Germany) using Soft Imaging software: *N* = 3 Control WT males, *N* = 5 ADAMTS-4 WT males, *N* = 8 Control SOD1^G93A^ males, *N* = 7 ADAMTS-4 SOD1^G93A^ males, *N* = 5 Control WT females, *N* = 3 ADAMTS-4 WT females, *N* = 5 Control SOD1^G93A^ females, *N* = 6 ADAMTS-4 SOD1^G93A^ females. Immunoreactivity for GFAP, Iba1, WFA or NGF were quantified using Image-Pro Plus software (Media Cybernetics, Rockville, MO, USA) at a pre-defined range, measured as the relative immunoreactive area for GFAP, Iba1, WFA or NGF. The number and size of ChAT-positive motoneurons were measured by using Image-Pro Premier software (Media Cybernetics). Co-localization of NeuN, GFAP or APC with ADAMTS-4 were assessed by a Zeiss LSM 700 confocal microscope coupled to a digital camera using Zen 2009 Image Analysis Software (Zeiss Inc., Maple Grove, USA).

### Primary neuron culture

Primary neuron cultures were prepared as described previously [49]. Cortices were isolated from 14 day-old mouse embryos in sterile Krebs solution containing 125 mM NaCl, 5 mM KCl, 1 mM NaH_2_PO_4_, 15 mM D-glucose, 25 mM HEPES, 0.05 mM BSA and 2 mM MgSO_4_. Cortices were then incubated for 15 min at 37 °C in Krebs solution containing 0.1 mM trypsin (Sigma-Aldrich). Krebs solution containing 25 nM DNAse (Sigma-Aldrich) and 130 nM soy bean trypsin inhibitor (Sigma-Aldrich) was added to the suspension (1:1 dilution), and then centrifuged at 250 g for 3 min. The pellet was resuspended in new Krebs solution, and centrifuged again. The cells were finally resuspended in Neurobasal medium supplemented with 2 % B-27 supplement and 0.5 mM L-glutamine. Neurons were plated on 48 well-plates at a density of 125 000 cells/well previously coated with 5 μg/ml poly-D-lysine and were used for experiments 6 days after plating. After 5 days, half of the medium was changed to complete Neurobasal medium.

### Treatment of neurons with recombinant proteins

Cultured neurons were treated with either a human recombinant ADAMTS-1 (2197-AD-020) or a human recombinant ADAMTS-4 (4307-AD-020; R&D SYSTEMS) at 20, 100, 200 or 500 ng/ml 30 min before exposure or not to 400 μM glutamate (Sigma-Aldrich) (*N* = 11-12 from 3 independent experiments).

### MTT assay

After 24 h’ exposure to glutamate, the neuron viability was assessed by measuring 3-(4,5-dimethylthiazol-2-yl)-2,5-diphenyltetrazolium bromide reduction (MTT; Sigma-Aldrich). For that purpose, cells were incubated with 120 μM MTT for 1 h before lysing in dimethyl sulfoxide (DMSO; J.T.Baker, Deventer, The Netherlands) and quantifying absorbance at 540 nm.

### Adult astrocyte culture

Primary cortical astrocyte cultures were prepared as described previously [50] with some modifications. Briefly, cortices were isolated from 6–8 week-old C57Bl/6 J mice and the tissue was suspended in Hank’s Balanced Salt Solution (HBSS, GIBCO, Life technologies) and centrifuged at 400 g for 5 min at RT. After the addition of 0.25 % trypsin-EDTA (GIBCO, Life technologies), the suspension was incubated at 37 °C for 30 min with occasional shaking. Fresh culture medium containing serum was added to neutralize the effect of trypsin and the suspension was centrifuged at 400 g for 5 min. The cells were treated with Percoll (Sigma-Aldrich) and centrifuged at 400 g for 10 min to separate the phases. The supernatant was discarded and the layer of glial cells was washed once with fresh culture media. The cells were plated onto poly-L-lysine coated flasks in Dulbecco’s Modified Eagle Medium Nutrient Mixture F-12 (DMEM/F12, GIBCO, Life technologies) containing 10 % heat-inactivated fetal bovine serum (FBS, GIBCO, Life technologies), 2 mM L-Glutamine (GIBCO, Life technologies), 100 U/ml penicillin/streptomycin (P/S, GIBCO, Life technologies) and G5 supplement (Invitrogen, Life technologies). The astrocytic culture contains on average 99 % of GFAP-positive cells. The microglial cells were removed by shaking at 200 g for 2 h prior to the experiments. Astrocytes were plated on 12 well-plates at a density of 50 000 cells/well and used for experiments 3 days after plating.

### Primary microglial culture

Primary microglial cultures were prepared as described previously [51, 52]. Brains were isolated from neonatal C57Bl/6 J mice (P1-2), washed in PBS containing 1 % glucose and mechanically and enzymatically dissociated using trypsin (TrypLE Express, GIBCO, Life technologies). The suspension was incubated at 37 °C for 20 min. Fresh culture medium containing FBS was added to neutralize the effect of trypsin. The suspension was plated in 15 cm-petradish in DMEM-F12 Glutamax (GIBCO, Life technologies) containing 1 % P/S and 10 % FBS (complete media). After 3 weeks, cells were shaken at 200 g and then washed with PBS before addition of 0.08 % trypsin-EDTA for 45 min in order to peel off the astrocytes. After removal of astrocytes, microglial cells were washed with PBS and 0.25 % trypsin-EDTA was added for 5 min. After neutralizing trypsin with complete media, cells were dislodged and centrifuged at 400 g for 5 min. Cells were resuspended in complete media. Microglia were plated on 24-well plates at a density of 200 000 cells/well. After one day, cells were placed in serum-free media and used for experiments 2 or 3 days after plating.

### Treatment of astrocytes and microglia with recombinant proteins

Cultured cortical astrocytes and neonatal microglia were treated with either a human recombinant ADAMTS-4 (4307-AD-020) or a human recombinant ADAMTS-1 (2197-AD-020; R&D SYSTEMS) at 20, 100 or 200 ng/ml for 48 h. The culture media were collected for ELISA assays (*N* = 3 in each group) and the RNAs (*N* = 4 in each group) were isolated from the corresponding cell layers for quantitative real-time PCR.

### siRNA assays

Silencing small-interfering RNAs (siRNAs) targeting the expression of ADAMTS-4 (Sigma-Aldrich) were transiently transfected in cultured cerebral microglia or cortical astrocytes with the lipofectamine 2000 reagent (Invitrogen, Life technologies) using the protocol provided by the manufacturer. For each well of a 12 well-plate, 2 μg of siRNA and 4 μl of lipofectamine were added to astrocytes previously deprived of serum. For each well of a 24 well-plate, 1 μg of siRNA and 2 μl of lipofectamine were added to microglial cells in serum-free fresh media. After 2 h of transfection, astrocytes or microglia were rinsed and fresh culture medium containing FBS (astrocytes) or not (microglia) was added. After 48 h, cells were rinsed with PBS and RNA/culture media were collected as described above. ADAMTS-4 siRNA sequences used were: Mm01-00044319, 5′-CCCAUAUCCUUGUACGGCA-3′ and 3′-UGCCGUACAAGGAUAUGGG-5′. As a control, empty vector (mock) was used (Mission siRNA Universal negative control #1, Sigma-Aldrich). ADAMTS-4 gene expression was significantly decreased by 69 % in astrocyte cultures (*N* = 4 in each group) and by 68 % in microglia cultures (*N* = 8 in each group) transfected with siRNA silencing ADAMTS-4 compared to mock.

### NGF ELISA

The NGF protein concentrations were measured in astrocyte (*N* = 3 and 4 in each group for respectively recombinant and siRNA experiments) or microglia (*N* = 4 and  5 in each group for respectively recombinant and siRNA experiments) culture media using the *ChemiKine*^TM^ NGF sandwich ELISA (Merck Millipore) following the manufacturer’s instructions.

### Statistical analyses

The data are expressed as mean ± SEM. An alpha level of *P* < 0.05 was used for determination of significance in all statistical tests. Molecular and cellular statistical analyses were performed with the Statview software package (v5.0). Kruskal-Wallis test was used for intergroup multiple comparisons. In significant cases, Mann–Whitney *U*-test was applied as post hoc test. Behavior and immunohistochemistry statistical analyses were performed using GraphPad Prism 5 (GraphPad Software Inc.). Kaplan-Meier survival analyses and log-rank test were used to compare the symptom onset of untreated SOD1^G93A^ and ADAMTS-4-treated SOD1^G93A^ mice. Two-way ANOVA was used to compare anatomical differences between genotypes. Unpaired two-tailed t-test was used for multiple comparisons.

## Abbreviations

AD: Alzheimer disease
ADAMTS: a disintegrin and metalloproteinase with thrombospondin motifs
ALS: amyotrophic lateral sclerosis
APC: Adenomatous polyposis coli
BDNF: brain-derived neurotrophic factor
BSA: bovine albumin serum
ChAT: choline acetyltransferase
CNS: central nervous system
CREB: cAMP response element-binding protein
CSPG: chondroitin sulfate proteoglycan
DMEM: Dulbecco’s Modified Eagle Medium
DMSO: dimethyl sulfoxide
ECM: extracellular matrix
EDTA: ethylene diamine tetraacetic acid
EGTA: ethylene glycol tetraacetic acid
ES: end stage
FBS: fetal bovine serum
FDA: Food and Drug Administration
GAPDH: glyceraldehyde-3-phosphate dehydrogenase
GDNF: glial cell-derived neurotrophic factor
GFAP: glial fibrillary acidic protein
HBSS: Hank’s Balanced Salt Solution
HDAC6: histone deacetylase
HPLAN1: hyaluronan and proteoglycan link protein 1
Iba1: ionized calcium-binding adapter molecule-1
KO: knock-out
miR: Micro-RNA
MOM: mouse on mouse
MTT: 3-(4,5-dimethylthiazol-2-yl)-2,5-diphenyltetrazolium bromide reduction
NF-κB: nuclear factor kappa b
NGF: nerve growth factor
NGS: normal goat serum
NMJ: neuromuscular junction
NRS: normal rabbit serum
OCT: Optimal Cutting Temperature
p75NTR: tumor necrosis factor receptor
PB: phosphate buffer
PBS: phosphate buffered saline
PFA: Paraformaldehyde
PNN: perineuronal net
PS: presymptomatic stage
RNA: ribonucleic acid
RT: room temperature
RT-PCR: real time polymerase chain reaction
SC: spinal cord
SCI: spinal cord injury
SEM: standard error of the mean
siRNA: small interference silencing RNA
SOD1: superoxide dismutase 1
SS: symptomatic stage
TDP43: TAR DNA-binding protein 43
TIMP-3: type 3 tissue inhibitor of metalloproteinases
TrkA: tyrosine kinase receptor A
WFA: Wisteria Floribunda Agglutin
WT: wild-type
