# Erratum to: ADAMTS-4 promotes neurodegeneration in a mouse model of amyotrophic lateral sclerosis

**DOI:** 10.1186/s13024-016-0080-9

**Published:** 2016-02-08

**Authors:** Sighild Lemarchant, Yuriy Pomeshchik, Iurii Kidin, Virve Kärkkäinen, Piia Valonen, Sarka Lehtonen, Gundars Goldsteins, Tarja Malm, Katja Kanninen, Jari Koistinaho

**Affiliations:** Department of Neurobiology, A. I. Virtanen Institute for Molecular Sciences, Biocenter Kuopio, University of Eastern Finland, Kuopio, 70211 Finland

Unfortunately, after publication of this article [1], an error was discovered in Fig. 12 (Fig. 1 here) that was introduced during the Production process. The corrected figure can be seen below and the original article has also been updated to reflect this change.

**Fig. 1:**
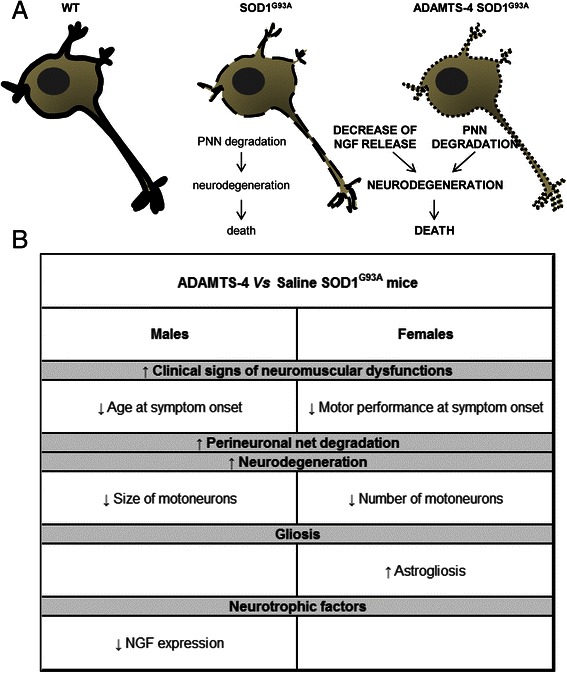
Gender similarities and differences in the effect of ADAMTS-4 treatment on ALS. **a** Schematic representation of ADAMTS-4 treatment promoting the decline of NGF production and ALS-induced perineuronal net degradation which contribute to the degeneration and even death of motoneurons in the ventral horn of the lumbar spinal cord of SOD1G93A mice. **b** A table describing the similarities and differences observed in behavioral and anatomical effects of ADAMTS-4 treatment in SOD1G93A male and female mice
